# Radiation-Induced Modification of Antioxidant-Active Biodegradable Starch–PVA Films: Effect of Phenolic Additive Structure and Nanofiller Reinforcement

**DOI:** 10.3390/polym18212619

**Published:** 2026-10-22

**Authors:** Betga Alex Worldlight, Krystyna Cieśla, Wojciech Głuszewski

**Affiliations:** Institute of Nuclear Chemistry and Technology, 03-195 Warsaw, Poland

**Keywords:** starch–PVA films, gallic acid/propyl gallate, gamma and electron-beam irradiation, cellulose nanocrystals

## Abstract

Starch-based films containing natural antioxidants are of interest for active food packaging, but the combined effects of reinforcement, phenolic structure and radiation processing remain underexplored. Starch–poly(vinyl alcohol) (PVA) films prepared from gamma-pre-irradiated starch, containing cellulose nanocrystals (CNCs, 1%), sodium alginate (5%) or both, and loaded with gallic acid (GA) or propyl gallate (PG), were irradiated with γ-rays or electron beams at 5 and 10 kGy. Tensile properties, swelling, solubility, insoluble fraction, contact angle, thermal behaviour, antioxidant capacity of ethanol extracts and gas evolution were measured. Formulation and additive effects were interdependent. PG increased tensile strength in the unfilled, alginate and CNC films (up to 16.88 ± 0.66 MPa for starch–PVA–alginate–PG), whereas the CNC/alginate/GA film combined 14.36 ± 0.61 MPa with an elongation at break of 506 ± 20%. CNC/alginate films had the highest insoluble fraction before irradiation (about 54%). Irradiation left tensile properties unchanged in most formulations but reduced the insoluble fraction by up to 24% in six of the eight films and lowered swelling mainly in CNC-containing films, indicating a net loss of retained material; crosslinking and chain scission were not measured directly. All films remained hydrophilic (contact angles 55–70°). The antioxidant capacity of the ethanol extracts changed little with irradiation, and H_2_ and O_2_ yields were higher in PG than in GA films. The mechanisms behind these observations were not tested. Barrier, migration, microstructural, biodegradation and food-preservation properties were not evaluated.

## 1. Introduction

The extensive use of petroleum-derived plastics in food packaging has become a major environmental concern because they degrade slowly, accumulate as waste, and contribute to microplastic pollution in soil and aquatic ecosystems [1,2,3,4,5]. These concerns have accelerated research into biodegradable, bio-based polymer films that can replace conventional plastics while offering additional functional benefits, such as antioxidant or antimicrobial activity, for active food packaging applications [6,7,8,9,10]. Starch is among the most promising candidates for such materials because of its natural abundance, low cost, biodegradability, and film-forming capacity [3,11,12,13]. However, starch films alone typically have poor mechanical strength, are highly moisture-sensitive, and tend to undergo retrogradation and embrittlement over time, limiting their use as standalone packaging materials [11,12,13,14].

To address these limitations, researchers have often combined starch with poly(vinyl alcohol) (PVA), a biodegradable synthetic polymer that forms high-quality films, offers flexibility, and readily bonds with starch through hydrogen bonding [15,16,17]. Compared with starch alone, starch–PVA blends show improved mechanical integrity and processability [18]. Researchers widely incorporate cellulose nanocrystals (CNCs) to further strengthen these matrices. Their high crystallinity, large surface area, and strong interfacial interactions with polysaccharide chains increase tensile strength and reduce water vapour permeability [17,19,20]. Sodium alginate has also been used as a natural anionic polysaccharide to modulate crosslinking density, water resistance, and film flexibility through interactions with the starch–PVA network [21,22,23,24,25,26]. In parallel, researchers have incorporated natural phenolic compounds such as gallic acid (GA) [27,28,29,30] and its lipophilic ester derivative, propyl gallate (PG) [31,32], into biopolymer films to confer antioxidant activity, an essential property for protecting oxidation-sensitive or lipid-rich foods during storage. Ionising radiation processing has also gained attention as a green, additive-free method for inducing crosslinking in starch–PVA matrices. It improves gel fraction, mechanical stability, and moisture resistance without chemical crosslinking agents [33,34,35,36].

Despite advances, prior studies have examined CNC reinforcement, alginate incorporation, phenolic antioxidants, and radiation crosslinking mostly in isolation or partial combinations. Side-by-side comparisons of CNC and alginate, individually and together, in radiation-processed starch–PVA films remain scarce, particularly regarding physicochemical, mechanical, and antioxidant properties. Although gallic acid (GA) and propyl gallate (PG) differ in polarity and lipophilicity, which may affect matrix compatibility, migration, and radical-scavenging efficiency, their performance has not been directly compared in the same radiation-crosslinked starch–PVA–CNC–alginate system. It is also unclear whether radiation preserves, enhances, or diminishes their antioxidant activity after incorporation and crosslinking. To our knowledge, this is the first systematic evaluation of CNC and alginate reinforcement with two chemically distinct phenolic antioxidants (GA and PG) in radiation-processed starch–PVA films. By separating nanofiller reinforcement and biopolymer crosslinking from antioxidant chemistry, the approach clarifies how they interact. The films combine mechanical strength, controlled swelling, tunable hydrophilicity, and sustained antioxidant activity. This scalable, additive-free radiation process integrates renewable nanoreinforcement and natural antioxidants, offering a metric-driven framework for designing biodegradable, antioxidant-active starch films. The findings support reducing conventional plastic packaging and extending food shelf life.

## 2. Materials and Methods

### 2.1. Materials

Cornstarch (Sigma S4126; amylose 27%) and fully hydrolysed poly(vinyl alcohol) (Merck; Mw = 145 kDa; degree of hydrolysis 99%) were used. The starch was pre-irradiated with gamma rays (10 kGy, 5 kGy/h). Sodium alginate, C_6_H_9_NaO_7_ (Biomus, cat. no. 9005-38-3), and hydrophilic crystalline nanocellulose (CNC) powder were obtained from the Process Development Centre at the University of Maine and produced by hydrolysis with sulphuric acid. Glycerol (Chempur; 98%), gallic acid (Thermo Scientific; anhydrous; 170.12 g/mol), and propyl gallate (Thermo Scientific; 98%; 212.20 g/mol; C_10_H_12_O_5_) were used. Antioxidant assays used 6-hydroxy-2,5,7,8-tetramethylchroman-2-carboxylic acid (Trolox; Sigma 53188-07-01) and DPPH (Aldrich Sigma). Ethanol (96%) and deionised water were also used.

### 2.2. Experimental Methods

#### 2.2.1. Film Preparation

Starch (gamma-pre-treated, 10 kGy), PVA, glycerol, and, where applicable, CNC and sodium alginate were mixed with water in the proportions specified in [11], which are listed in Table 1. After the mixture cooled to 45 °C, PG or GA (3.45% w/w of total polymer mass; 0.69 g per 720 mL batch) was dissolved in 5 mL of ethanol/solvent and added dropwise while homogenising (25,000 rpm for 5 min). The mixture was then stirred for 5 min. The film-forming solution was cast into 12 Petri dishes (10 cm; 60 mL each) and dried at 17 °C and 43% RH for 48–72 h. The films were 150–160 µm thick.

#### 2.2.2. Irradiation

The films were irradiated at room temperature under air atmosphere using fast electrons or gamma rays (^60^Co). Gamma irradiation was performed in a GC 5000 gamma chamber (Institute of Nuclear Chemistry and Technology, Warsaw, Poland) equipped with a ^60^Co source operating at a dose rate of 1.1 kGy/h, verified by Fricke dosimetry before testing. The self-shielded chamber has a central irradiation cavity surrounded by an array of ^60^Co pencil sources. Samples were stacked in the cavity, and Fricke dosimetry confirmed dose uniformity, with a ratio of Dmax/Dmin = 1.1. Electron-beam irradiation was conducted using an Elektronika 10/10 linear accelerator operating at 10 MeV and approximately 14,000 kGy/h; the dose rate and uniformity were also verified by Fricke dosimetry. Samples were placed on the standard conveyor beneath the scanning horn and irradiated on both sides to ensure an even dose.

### 2.3. Mechanical Test

Mechanical testing followed ASTM D882 (Standard Test Method for Tensile Properties of Thin Plastic Sheeting) on an Instron 5565 universal testing machine at 20 mm/min, with a 50 mm gauge length. Film thickness was measured at three locations using a digital micrometre. Specimens (60 × 10 mm) were tested to failure; tensile strength and elongation at break were obtained from stress–strain curves. Eight replicates were tested per composition.

### 2.4. Swelling Behaviour

Square film specimens (10 × 10 mm) were weighed, immersed in distilled water at 25 °C for 24 h, blotted dry, and weighed again. They were then dried at 110 °C to a constant mass. The mass change relative to the initial dry mass was used to calculate the swelling parameters (A and B), expressed as percentages, according to the specified equations.

(1) $$swelling\;\left(Initial\right)\;A\;=\;100\frac{W_{s}-W_{0}}{W_{0}}$$

(2) $$Swelling\;(dried)\;B\;=\;100\frac{W_{s}-W_{d}}{W_{d}}$$

### 2.5. Insoluble Fraction

Sample portions (approximately 150 mg) were placed in pre-weighed tubes containing water and heated with stirring at 100 °C for 2 h. After cooling, the liquid was separated from the gel, which was then dried at 110 °C to a constant weight. The gel percentage was calculated from three measurements using the formula.

(3) $$Gel\;fraction=100\frac{W_{g}}{W_{g}+W_{s}}$$

W_g_ is the dry mass of the insoluble (gel) fraction retained after water treatment; and W_s_ is the dry mass of the soluble fraction recovered from the liquid. Their sum (W_g_ + W_s_) is the total recovered dry mass and should match the sample’s initial dry mass within experimental mass-balance tolerance.

### 2.6. Contact Angle

To assess film hydrophilicity or hydrophobicity, we measured water contact angles at the INCT Laboratory of Materials Research. A microsyringe was used to place a 5 µL droplet on each film’s air-side surface; images were captured after 10 s. Measurements were taken at 25 °C and 40–50% relative humidity. For each composition, at least six samples were tested, with five measurements per sample (30 total per composition). The wetting angle was calculated using the following equation:

(4) $$\theta =arc\cos\frac{1-({h/r)}^{2}}{1+({h/r)}^{2}}$$

where h and r denote the height and radius of the enlarged drop image.

### 2.7. Gas Chromatography

Radiolytic H_2_ and O_2_ were determined for films irradiated with γ-rays (^60^Co, 1.1 kGy/h) at 28 kGy. Samples (1.0 g) were placed in 5 mL glass vials, sealed with a septum under air, and irradiated in the vials. Headspace gas (100 µL) was withdrawn with a gas-tight syringe and analysed on a Shimadzu GC-2014 with a thermal conductivity detector and a 5A molecular sieve column. Analyses were performed immediately after irradiation. The studies covered gamma radiation exposure. Analyses were performed in triplicate, and the mean value was calculated. Preliminary calibration for hydrogen and oxygen determination was performed. Based on the analysis results, the number of moles of radiolytically evolved hydrogen was calculated. The energy value was calculated as the product of the sample mass and the dose expressed in Gy (J/kg). Dividing the two values yielded the radiolytic yield in µmol/J.

Formula for hydrogen determination

(5) $$G\left(µmol/J\right)=\frac{A\times \left(5000-1000\right)}{\left(33898\times 100\times 1\times 22.4\times 28\right)}$$

where A: analysis result; 5000: vial volume; 1000: polymer volume; 33,898: calibration result; 100: syringe volume; 1: sample mass; and 28: dose in kGy.

### 2.8. Statistical Analysis

All quantitative results are reported as the mean ± standard deviation. Tensile properties (tensile strength and elongation at break) were measured using eight specimens per formulation (n = 8). Swelling, solubility, and gel fraction were measured in triplicate (n = 3). Contact angle values are reported as the mean of 30 droplet measurements across at least six independent films, while gas evolution was measured in duplicate (n = 3). For tensile, swelling/solubility, and gel fraction data, differences among the four formulations were assessed under each additive and irradiation condition using one-way analysis of variance (ANOVA), followed by Tukey’s HSD post hoc test. Each formulation came from a single casting batch, and the specimens are subsamples of that batch. Irradiation effects (dose and modality) were assessed within each formulation using two-way ANOVA. Phenolic additive effects were assessed within each formulation using two-way ANOVA (additive × irradiation condition; 0 kGy, 10 kGy γ, and 10 kGy EB), considering only conditions common to loaded and unloaded films. Non-additivity of CNC and alginate was tested using the CNC × alginate interaction, and its dependence on additive and irradiation was assessed using higher-order interactions. Additive comparisons were adjusted across the six formulations using the Holm procedure; a significant additive × irradiation interaction was interpreted as a dose-dependent additive effect. Differences were considered statistically significant at *p* < 0.05.

### 2.9. Thermogravimetric Analysis

Thermogravimetric analysis was performed using a Thermobalance TGA Q500 V20.7 (TA Instruments) under nitrogen flowing at 60 mL/min. Approximately 15 mg of each sample was heated from 30 °C to 600 °C at 10 °C/min. Each value comes from a single run. The onset degradation temperature (T_0_) was determined from the intersection of tangent lines drawn before and after the onset of the degradation step. The peak degradation temperature (T_p_) was defined as the temperature at which the first derivative thermogravimetric (DTG) curve reached its maximum mass-loss rate. The final degradation temperature (T_f_) was defined as the temperature corresponding to the residual mass percentage.

### 2.10. Antioxidant Activity

The antioxidant activity of film extracts was evaluated using the DPPH and TEAC assays, which also monitored gallic acid release from the polymer over time. A 25 mg film sample was immersed in ethanol and incubated under controlled conditions. Extractions were performed at 5, 10, 96, and 672 h to capture the initial burst, diffusion-controlled, and long-term equilibrium release phases.

Trolox calibration curves (0–250 µmol/L; R^2^ > 0.99) were used to determine TEAC, with results reported as µmol Trolox equivalents per gram of dry film. DPPH radical-scavenging activity was evaluated by calculating percentage inhibition relative to the control solution.

Appropriate blanks (without extract) and solvent controls were used to correct for background absorbance and to verify that the antioxidant activity mainly arose from gallic acid release. The mixture was kept in the dark for 30 min before absorbance was measured at 517 nm. Radical-scavenging activity was evaluated using the DPPH method, with the film extract treated as the blank (A0). The percentage decrease in DPPH was calculated using the specified formula ([37]):

(6) $$\%\;reduction\;in\;DPPH\;reduction=\left(\frac{A_{0}-A_{t}}{A_{0}}\times 100\right)$$

A_0_ is the absorbance of DPPH;

A_t_ is the absorbance of the mixture of DPPH and the film’s extract.

The Trolox equivalent antioxidant capacity (TEAC) was determined using the second equation provided below

(7) $$TEAC\;(\mu mol/mg)\;=C(TE)/(C_{\left(film\;extract\right)}\times M_{\left(Trolox\right)})$$

## 3. Results

### 3.1. Mechanical Test

Tensile strength (TS) and elongation at break (EAB) depended on formulation and additive, with the effect of the additive varying across formulations (additive × formulation interaction: TS, F(3,56) = 53.3; EAB, F(3,56) = 188.8; both *p* < 0.001) (Table 2 and Table 3). The two-way ANOVA (formulation × irradiation condition) and planned contrasts are presented in Table 4 and Table 5. In PG films, formulation affected TS (F(3,28) = 8.9, *p* < 0.001; Table 2). S-P-A-PG exhibited the highest TS (16.88 ± 0.66 MPa), followed by S-P-C-PG (15.17 ± 1.51 MPa), S-P-C-A-PG (13.40 ± 1.74 MPa), and S-P-PG (12.46 ± 1.18 MPa). S-P-A-PG was stronger than S-P-PG and S-P-C-A-PG, while S-P-C-PG was stronger than S-P-PG (Tukey, *p* < 0.05). No other pairwise differences were significant, including comparisons between S-P-A-PG and S-P-C-PG, and between S-P-C-A-PG and S-P-PG. In GA films, S-P-C-A-GA had the highest TS (14.36 ± 0.61 MPa; F(3,28) = 58.1, *p* < 0.001). S-P-GA (9.30 ± 0.79 MPa) and S-P-C-GA (9.95 ± 0.99 MPa) did not differ significantly, while S-P-A-GA (11.29 ± 0.90 MPa) had an intermediate value. For S-P, S-P-A, and S-P-C, PG films had higher TS than the corresponding GA films by 3.2, 5.6, and 5.2 MPa, respectively (Holm-adjusted *p* < 0.001). For S-P-C-A, TS was similar between the two additives (GA: 14.36 ± 0.61 MPa; PG: 13.40 ± 1.74 MPa; difference not significant, *p* = 0.15). The stronger effect of alginate than CNC in PG films may result from the formation of numerous hydrogen bonds between alginate’s carboxyl groups and the hydroxyl groups of starch and PVA, thereby increasing intermolecular cohesion [27,28,29,30]. In contrast, CNC acts as a rigid filler, and its effect depends on its dispersion [31,32].

In GA films, neither CNC nor alginate alone improved performance, but combining the two did. This finding suggests that GA, with its three hydroxyl groups and one carboxyl group, may form hydrogen bonds with both fillers, helping to bring them together in a continuous, load-bearing network. This explanation remains hypothetical, however, because filler–additive interactions were not examined directly. The hydrophobic propyl ester group in PG reduces water uptake (Section 3.2), which may limit moisture-induced plasticisation and help preserve hydrogen bonding between polymer chains. In contrast, the more hydrophilic GA retains more water, leading to greater plasticisation of the matrix. This trend was reversed in the CNC/alginate system: S-P-C-A-GA was stronger than S-P-C-A-PG (*p* = 0.014). The hydrophobic PG ester may be less compatible when combined with CNC and alginate, potentially reducing filler dispersion and stress transfer. Further analysis, such as SEM, would be needed to confirm this possibility.

EAB followed a similar pattern. S-P-C-A-GA exhibited the highest EAB among all films (506.01 ± 19.93%; F(3,28) = 429.1, *p* < 0.001), substantially exceeding that of S-P-C-A-PG (274.05 ± 12.07%). In S-P-C-A, GA films were more extensible than PG films, whereas in S-P-C, PG films were more extensible (279.77 vs. 190.90%, *p* < 0.001). In S-P-A, EAB did not differ significantly between GA (359.84 ± 36.22%) and PG (325.16 ± 20.69%) (*p* = 0.076); likewise, the additives did not differ in S-P (*p* = 0.21). The highly hydrated alginate network, plasticised by GA, may facilitate chain sliding, while the CNC network bears the load and delays failure. This mechanism could explain the combination of high TS and high EAB observed in the film.

Irradiation did not significantly affect TS in PG films (condition F(4,140) = 0.40, *p* = 0.81; Table 4). In GA films, both the condition effect (F(4,140) = 5.55, *p* < 0.001) and the condition × formulation interaction (F(12,140) = 11.6, *p* < 0.001) were significant, driven by S-P-C-A-GA, whose TS decreased by 14–22% (14.36 to 12.40 MPa at 10 kGy γ, 11.77 MPa at 5 kGy EB and 11.26 MPa at 10 kGy EB; Holm-adjusted *p* < 0.001) and, to a smaller extent, by S-P-A-GA (11.29 to 10.20 MPa at 10 kGy γ; adjusted *p* = 0.018). EB-irradiated S-P-C-A-GA was weaker than the γ-irradiated film at both doses (Table 5). The remaining formulations showed no significant changes, suggesting that crosslinking and chain scission were approximately balanced.

The behaviour of the CNC/alginate films aligns with the gel fraction and gas evolution results (Section 3.3 and Section 3.5). Radiation degrades alginate through chain scission and decarboxylation, and alginate films produced the highest hydrogen yields. As an efficient radical scavenger, GA resulted in lower gel fractions than PG, suggesting that fewer crosslinks formed to counteract this scission. The strength loss in S-P-C-A-GA is therefore likely due to scission that was not offset by new crosslinks. Notably, the strongest film before irradiation was also the most affected. In S-P-C-A-PG, greater radical availability may promote crosslinking, but scission appears to have dominated at the highest dose rate and dose (10 kGy EB). EB irradiation at about 14,000 kGy/h produces a much higher radical density than gamma irradiation at 1.1 kGy/h. EAB increased significantly in S-P-A-PG (10 kGy EB: 325.16 to 374.24%, *p* < 0.001), S-P-C-PG (*p* < 0.001), S-P-C-A-PG (5 and 10 kGy γ and 10 kGy EB: about 6%, *p* = 0.041), and S-P-C-GA (10 kGy γ, *p* = 0.046; 5 kGy EB, *p* = 0.002). These small increases are consistent with limited chain scission, which shortens polymer chains and increases mobility without reducing TS. EAB remained unchanged in S-P-PG, S-P-GA, and S-P-A-GA (for the latter, the overall test was significant at *p* = 0.006, but no irradiated condition differed from the control after Tukey correction). In S-P-C-A-GA, EAB decreased after EB irradiation (at 5 kGy, *p* = 0.004; at 10 kGy, *p* < 0.001). This film lost both strength and extensibility after EB irradiation, supporting degradation of the CNC–alginate–GA network rather than increased mobility.

### 3.2. Swelling Behaviour

**Figure 1:**
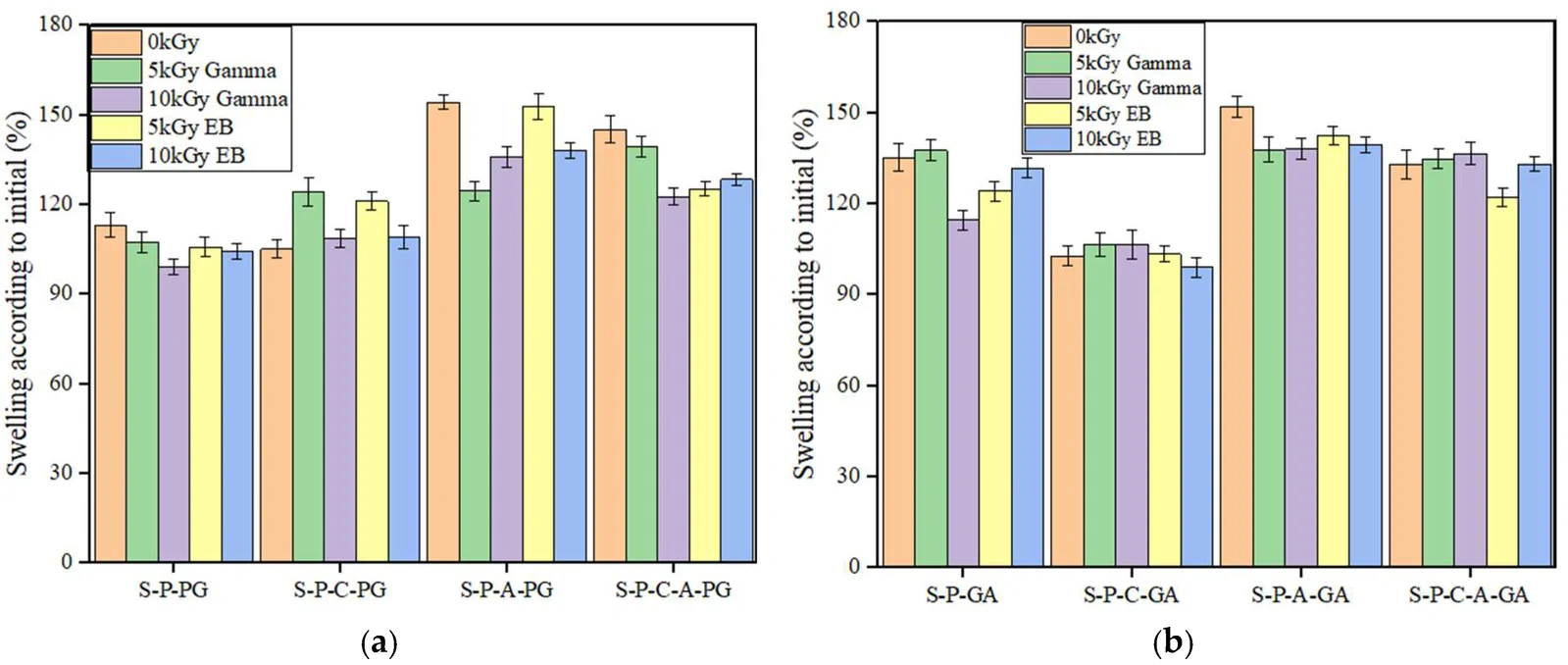
Swelling relative to initial mass (SW/INI, %) of (**a**) PG films and (**b**) GA films after 24 h in water at 25 °C.

**Figure 2:**
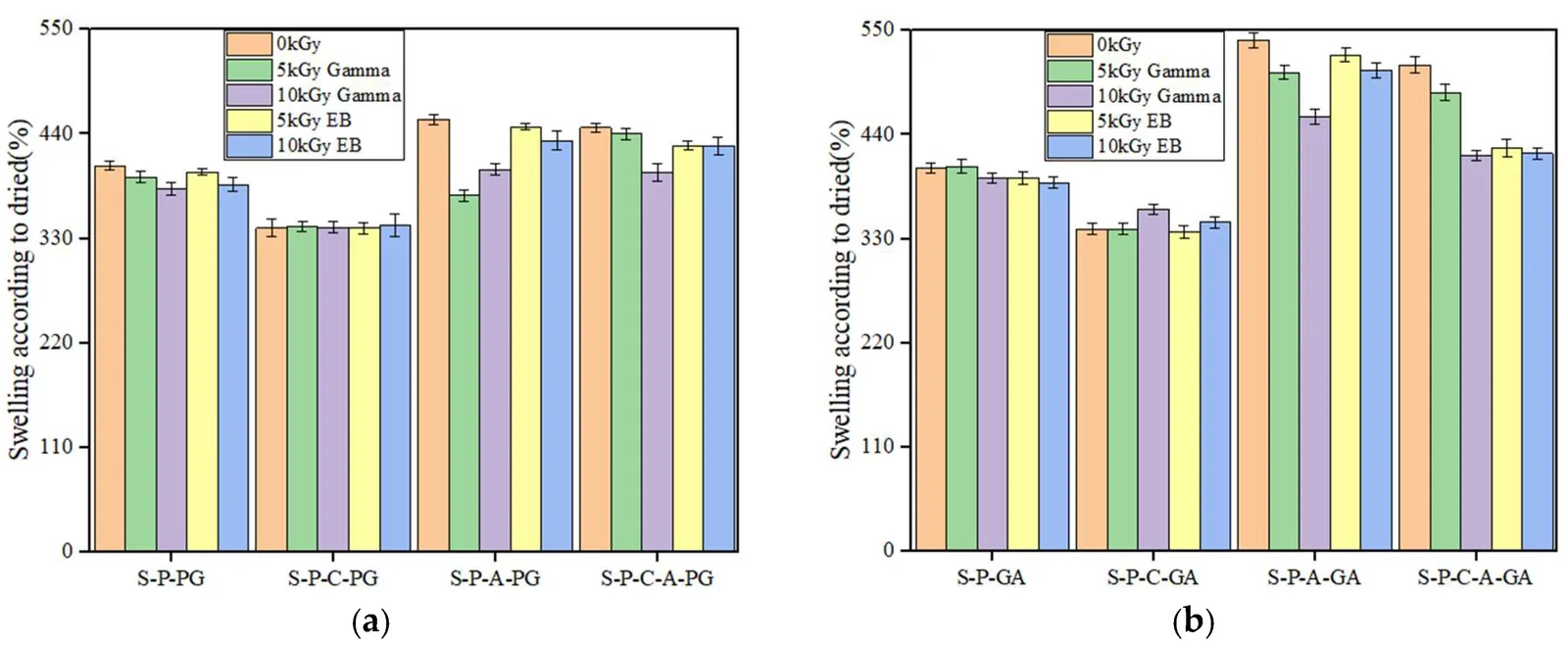
Swelling relative to dry mass (SW/Dried, %) of (**a**) PG films and (**b**) GA films.

**Figure 3:**
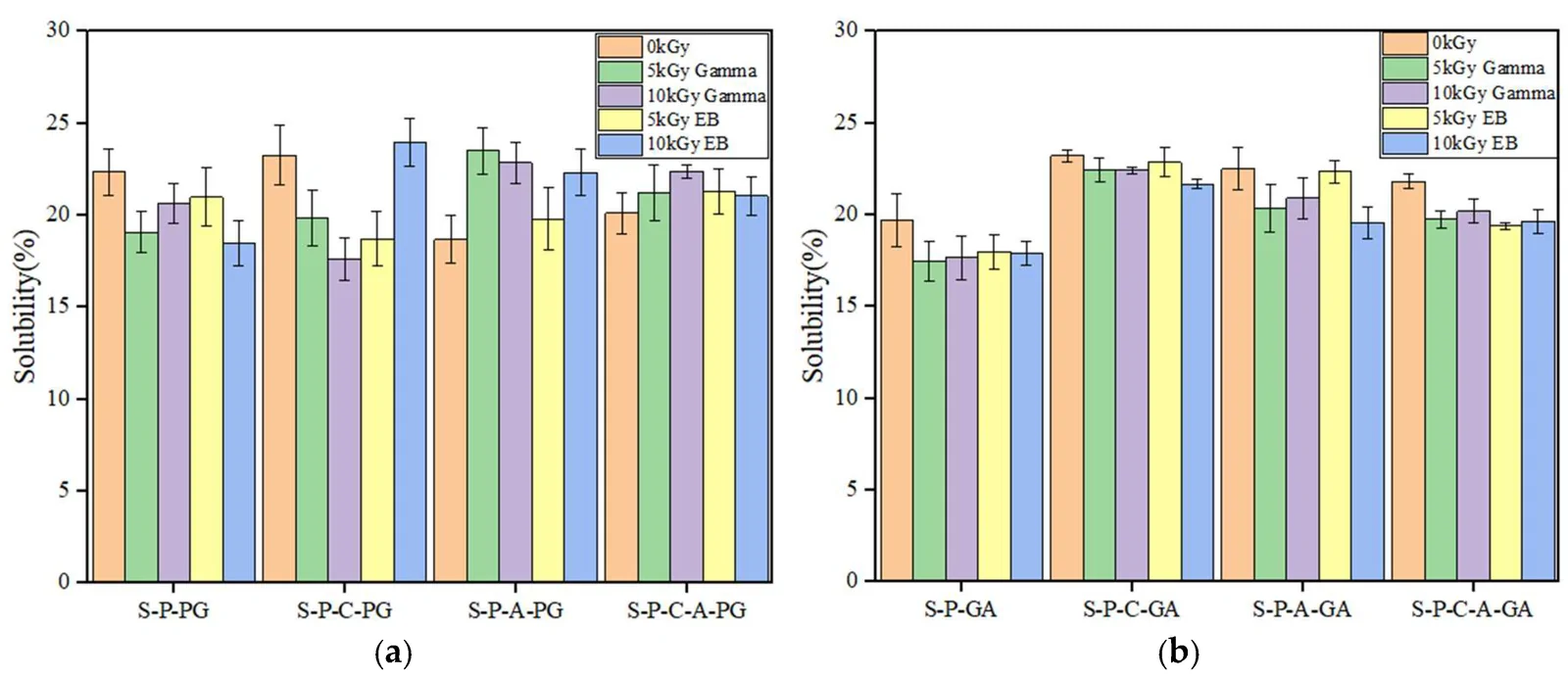
Solubility (%) of (**a**) PG films and (**b**) GA films.

Swelling and solubility were measured after 24 h in water at 25 °C (Figure 1, Figure 2 and Figure 3). At 0 kGy, formulation significantly affected all three responses in both series (one-way ANOVA, n = 3: SW/INI, PG F(3,8) = 59.6, GA F(3,8) = 48.9; SW/Dried, PG F(3,8) = 41.0, GA F(3,8) = 141.3; solubility, PG F(3,8) = 14.5, GA F(3,8) = 6.5; all *p* ≤ 0.015). CNC increased swelling: S-P-C-PG swelled more than S-P-PG (SW/INI 154.34 vs. 113.17%, *p* < 0.001; SW/Dried 455.21 vs. 406.45%, *p* = 0.020), and S-P-C-GA swelled more than S-P-GA (SW/INI 135.29 vs. 102.78%, *p* < 0.001; SW/Dried 404.55 vs. 340.38%, *p* < 0.001). The effect of alginate varied depending on the additive. In GA films, alginate increased swelling (S-P-GA to S-P-A-GA: SW/Dried 340.38 to 418.54%; SW/INI 102.78 to 133.01%; both *p* < 0.001). In PG films, alginate reduced SW/Dried (406.45 to 340.95%, *p* = 0.004), making S-P-A-PG the least-swelling PG film, but did not significantly affect SW/INI (113.17 to 105.15%, *p* = 0.33). Combining CNC and alginate produced the highest SW/Dried values in both series (PG, 466.72%; GA, 539.90%, the highest of all films). However, SW/INI for the S-P-C-A films did not differ significantly from that of the S-P-C films (*p* = 0.24 for PG, *p* = 0.11 for GA); overall, S-P-C-PG had the highest SW/INI (154.34%). The greater swelling of CNC films may be related to the nanocrystals’ hydroxyl- and sulphate-rich surfaces or to microchannels at the filler–matrix interface. The carboxyl groups of alginate and hydroxyl groups of GA may provide additional hydrophilic sites, which could help explain the alginate–GA result [33,34]; however, none of these explanations was tested. The film with the highest SW/Dried (S-P-C-A-GA) also had the highest EAB (506%, Section 3.1), while the PG film with the lowest SW/Dried (S-P-A-PG) had the highest TS (16.88 MPa). This pattern is consistent with a hydrated, plasticised network in the first case and a more cohesive network in the second. Swelling alone, however, does not explain the difference in TS between the additives: in S-P and S-P-C, PG films swelled more than GA films (below), yet were also stronger (Section 3.1).

Solubility was less sensitive to composition than swelling. Alginate did not significantly affect solubility (S-P vs. S-P-A: 22.35 vs. 23.27%, *p* = 0.65 for PG; 23.12 vs. 21.83%, *p* = 0.40 for GA). CNC reduced solubility (PG, 22.35 to 18.67%, *p* = 0.007; GA, 23.12 to 19.71%, *p* = 0.010), consistent with CNC restricting polymer-chain mobility and the leaching of soluble components [35,36,38,39,40]. Together, the lower solubility and higher swelling of CNC films indicate that these films took up more water while losing less material. Solubility did not differ between additives at 0 kGy (F(1,16) = 1.3, *p* = 0.28). Swelling did differ between additives, and the direction depended on alginate (GA minus PG, Holm-adjusted). SW/Dried was lower in GA than in PG films without alginate (S-P, −66.1 points; S-P-C, −50.7 points) and higher with alginate (S-P-A, +77.6 points; S-P-C-A, +73.2 points; all *p* < 0.001). SW/INI showed the same pattern, except that it did not differ in S-P-C-A (S-P, −10.4 points, *p* = 0.042; S-P-C, −19.1, *p* < 0.001; S-P-A, +27.9, *p* < 0.001; S-P-C-A, −0.2, *p* = 0.96). In films without alginate, GA might bridge starch and PVA chains via hydrogen bonds, whereas the bulkier, less polar PG might disrupt chain packing and increase free volume. These interactions were not measured, so this remains a hypothesis.

Irradiation affected swelling differently depending on the formulation (formulation × irradiation interaction, *p* < 0.001 for SW/INI and SW/Dried in both series; comparisons with the 0 kGy film are Holm-adjusted). In PG films, SW/Dried decreased in S-P-C (−80 and −53 points, i.e., −17.6% and −11.6%, at 5 and 10 kGy γ; *p* ≤ 0.004; not after EB) and in S-P-C-A (−67 points, −14.3%, at 10 kGy γ; −39 points, −8.3% and −8.4%, at 5 and 10 kGy EB; *p* ≤ 0.035), but not in S-P (*p* = 0.26) or S-P-A (*p* = 10). In GA films, SW/Dried decreased significantly only in S-P-C-A at 10 kGy γ (−81 points, −15.0%, *p* < 0.001). SW/INI decreased in S-P-C-PG (−30 points, −19.3%, at 5 kGy γ; −18 and −16 points at 10 kGy γ and EB; *p* ≤ 0.013), in S-P-C-A-PG (−22, −20 and −17 points at 10 kGy γ, 5 kGy EB and 10 kGy EB, i.e., −11.6 to −15.5%; *p* ≤ 0.013), and in S-P-PG at 10 kGy γ only (−14 points, −12.5%, *p* = 0.013). In contrast, SW/INI of S-P-A-PG increased at 5 kGy γ and 5 kGy EB (+19 and +16 points, +18% and +15%; *p* ≤ 0.011). In GA films, SW/INI decreased only in S-P-C (−21 points, −15.2%, at 10 kGy γ; −11 points, −8.2%, at 5 kGy EB; *p* ≤ 0.041). A decrease in swelling may result from crosslinking, which limits chain mobility and water uptake [41,42,43,44,45]. However, crosslink density was not measured, and the insoluble fraction decreased after irradiation (Section 3.3), so changes in the amount of material lost or in physical associations could produce similar effects. The stronger response of CNC-containing films may suggest that the nanocrystals contribute to the radiation response, although this was not tested. The absence of a swelling decrease in S-P-A-PG, whose SW/INI increased while its solubility decreased, may reflect less material being lost, although the insoluble fraction of this film did not change after hot-water treatment (Section 3.3). S-P-C-A-GA swelled less after 10 kGy γ (Section 3.2) and lost tensile strength after all irradiation conditions and extensibility after EB irradiation (Section 3.1); these changes, however, did not occur under the same conditions.

Solubility responded to irradiation differently in the two series (formulation × irradiation interaction: PG, *p* < 0.001; GA, *p* = 0.43). In PG films, solubility decreased in S-P (by 6–17%, *p* ≤ 0.024, in all irradiated conditions) and in S-P-A (by 15%, 24% and 20% at 5 kGy γ, 10 kGy γ and 5 kGy EB; *p* < 0.001; not at 10 kGy EB). This may indicate that polymer was retained in the network, but it was not examined directly. In contrast, solubility increased in S-P-C-PG (by 26%, 22% and 20% at 5 kGy γ, 10 kGy γ and 10 kGy EB; *p* ≤ 0.001) and in S-P-C-A-PG at 10 kGy γ only (by 11%, *p* = 0.002), even though swelling decreased in these films. This pattern is consistent with crosslinking and chain scission occurring together: a denser network that swells less and low-molecular-weight fragments that can be extracted, but no measurement here separates these processes. In GA films, solubility decreased modestly in S-P-A under all four irradiated conditions (7.5–11%, *p* ≤ 0.002) and in S-P at 10 kGy EB (6%, *p* = 0.024), and did not change significantly in S-P-C or S-P-C-A after correction. The two series responded differently, but because no antioxidant-free films were examined, we cannot attribute the difference to the radical-scavenging activity of GA or PG. EB and γ irradiation differed at the same dose only in some films, in both directions and without a consistent pattern (for example, SW/Dried of S-P-C-PG at 5 kGy was 73 points higher after EB than after γ, *p* < 0.001; solubility of S-P-A-PG at 10 kGy was 6.4 points higher after EB, *p* < 0.001; SW/Dried of S-P-C-A-GA at 10 kGy was 49 points higher after EB, *p* = 0.009) [46,47,48].

### 3.3. Insoluble Fraction

**Figure 4:**
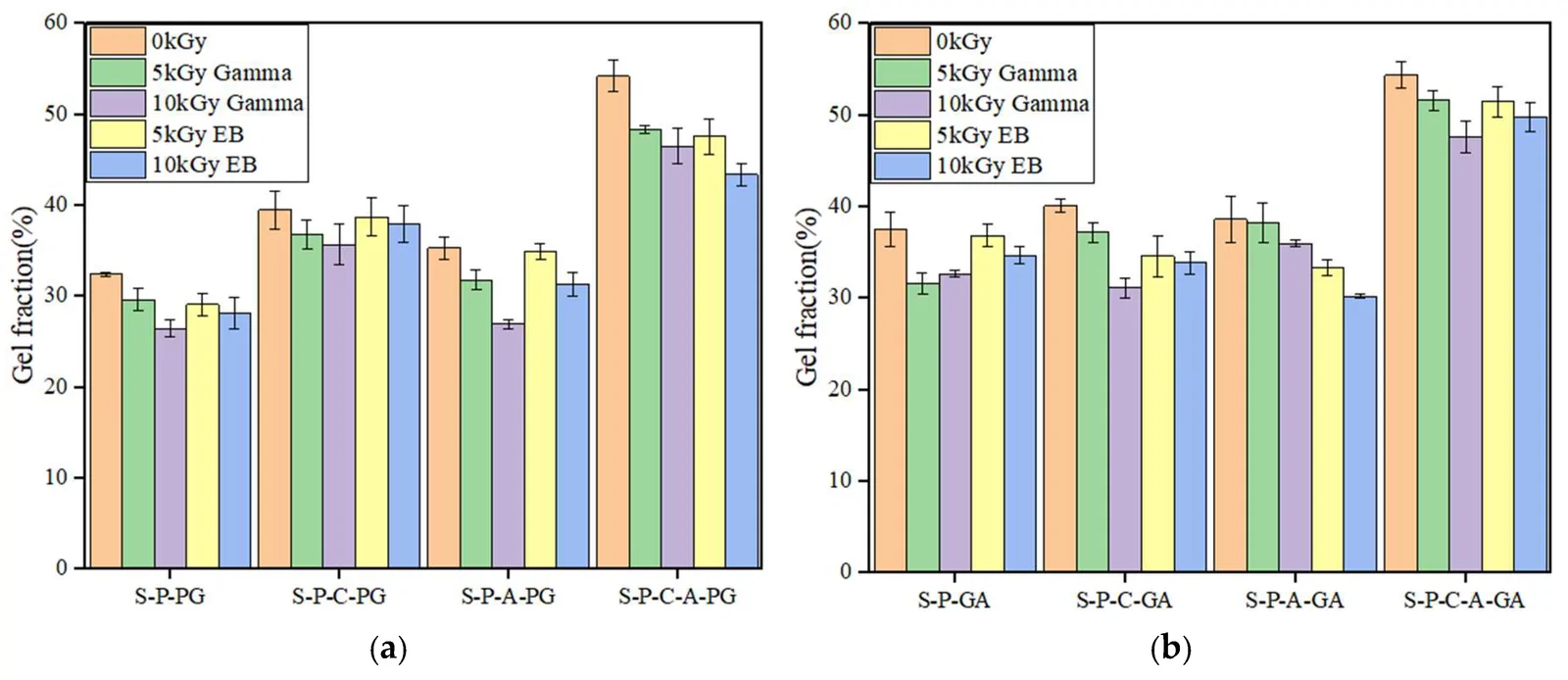
Gel fraction (%) of PG films (**a**) and GA films (**b**) before and after irradiation.

Formulation had a significant effect on the insoluble fraction in non-irradiated films containing either additive (PG, F(3,8) = 67.9; GA, F(3,8) = 61.1; both *p* < 0.001; Figure 4). The highest values were observed in films containing both fillers (S-P-C-A-PG and S-P-C-A-GA), about 14–22 points higher than in all other formulations (*p* < 0.001). In PG films, adding alginate increased the insoluble fraction compared with S-P (*p* = 0.012), while adding CNC alone did not (*p* = 0.36). In GA films, neither alginate nor CNC alone differed from S-P (*p* ≥ 0.35). The substantial increase when CNC and alginate were combined suggests a possible synergistic effect. One possible explanation is that CNC, which on its own may be present at too low a level to form a continuous network, interacts with alginate’s carboxyl and hydroxyl groups through hydrogen bonding. Together with starch and PVA, these interactions may form a percolating, water-insoluble physical network [49,50,51]. Since this fraction was present at 0 kGy, it cannot be attributed to irradiation. The high tensile strength and elongation at break of S-P-C-A-GA (Section 3.1) are consistent with such a network, although they do not confirm that one formed. CNC is itself insoluble, so some of the insoluble fraction in CNC-containing films consists of retained filler. However, CNC makes up only 1% of the polymer solids, so retained filler can account for at most about one percentage point. The composition of the insoluble fraction was not analysed.

At 0 kGy, GA films had insoluble fractions that were equal to or higher than those of PG films (additive effect, F(1,16) = 8.7, *p* = 0.010; additive × formulation interaction, F(3,16) = 4.0, *p* = 0.027). This difference was significant only for S-P-C (40.08 vs. 35.33%, Holm-adjusted *p* = 0.031; *p* ≥ 0.12 for other formulations). After irradiation, GA films generally retained higher values (additive effect: 5 kGy γ, *p* < 0.001; 10 kGy γ, *p* < 0.001; 10 kGy EB, *p* = 0.036; 5 kGy EB, not significant, *p* = 0.084), particularly in S-P and S-P-C. For example, at 10 kGy γ and 5 kGy EB, PG films in S-P were 6.2 and 7.7 points lower, respectively (*p* = 0.010 and 0.006). GA has three hydroxyl groups and one carboxyl group, so it may form more hydrogen bonds between polymer chains than PG, helping preserve a larger insoluble physical network. This interpretation is consistent with the lower swelling of S-P-GA and S-P-C-GA compared with the corresponding PG films (Section 3.2). However, this remains a hypothesis, as these interactions were not measured directly.

Irradiation reduced the insoluble fraction. The overall effect was significant for both additives (PG, F(4,40) = 22.1; GA, F(4,40) = 17.9; both *p* < 0.001), and the extent of the reduction varied by formulation (interaction, *p* = 0.005 for PG and *p* < 0.001 for GA). No formulation showed a significant increase. In PG films, the insoluble fraction decreased in S-P (10 kGy γ, −18%, *p* = 0.005; 10 kGy EB, −13%, *p* = 0.035), S-P-C (5 kGy γ, −10%; 10 kGy γ, −24%; 10 kGy EB, −11%; *p* ≤ 0.011), and S-P-C-A (−11 to −20% across all irradiated conditions, *p* ≤ 0.018), but remained unchanged in S-P-A (F(4,10) = 1.1, *p* = 0.42). In GA films, it decreased in S-P (5 and 10 kGy γ, −16 and −13%; *p* ≤ 0.004), S-P-A (5 and 10 kGy EB, −14 and −22%; *p* = 0.036 and 0.002), and S-P-C (10 kGy γ, −22%, *p* < 0.001; EB, −14 to −16%, *p* ≤ 0.022). In S-P-C-A-GA, the decrease was not statistically significant (−5 to −12%; overall test *p* = 0.085).

The decrease indicates a net loss of retained material at 5–10 kGy. This may reflect starch and alginate chain scission, which occurs mainly through glycosidic bond cleavage, as well as the low efficiency of PVA crosslinking at these doses, allowing soluble fragments to be extracted. It may also result from disruption of physical associations. Phenolic antioxidants (PG and GA) may scavenge macroradicals and thereby limit covalent crosslink formation. However, because we did not examine antioxidant-free (unloaded) films, we cannot distinguish their contribution to the radiation response from that of the polymer matrix itself. Accordingly, we describe the decrease in the insoluble fraction for the loaded films as a whole. Differences between GA and PG films are treated as differences between the additives, not as evidence of scavenging. Because the starch was pre-irradiated (Section 2.1), the response may also partly reflect a starch component that was already depolymerised; the findings should not be extended to films made from native starch.

The insoluble fraction agrees only partly with the solubility measured in Section 3.2. Solubility increased in S-P-C-PG (three irradiated conditions) and in S-P-C-A-PG (10 kGy γ), films that also showed large declines in insoluble fraction, and tensile strength decreased in the CNC/alginate films (Section 3.1). However, solubility decreased after irradiation in S-P-PG, whose insoluble fraction also decreased, and in S-P-A-PG, whose insoluble fraction did not change. The lower swelling of CNC-containing films after irradiation (Section 3.2) also does not contradict a net loss of material: the insoluble fraction measures retained polymer, whereas swelling depends on the structure of the remaining network. A smaller, denser, or more compact residual network might swell less, but crosslink density was not measured and cannot be inferred from these data. After irradiation, S-P-C-A films retained the highest insoluble fractions (approximately 43–52%; the maximum of 54.38% is the non-irradiated value), consistent with a predominantly physical network that was less affected by irradiation. Molecular-weight analysis of the soluble fraction, spectroscopic analysis, and crosslink-density measurements would be needed to distinguish chain scission and covalent crosslinking from changes in physical association. Unloaded controls irradiated under the same conditions would be needed to isolate the effects of GA and PG.

### 3.4. Contact Angle

**Figure 5:**
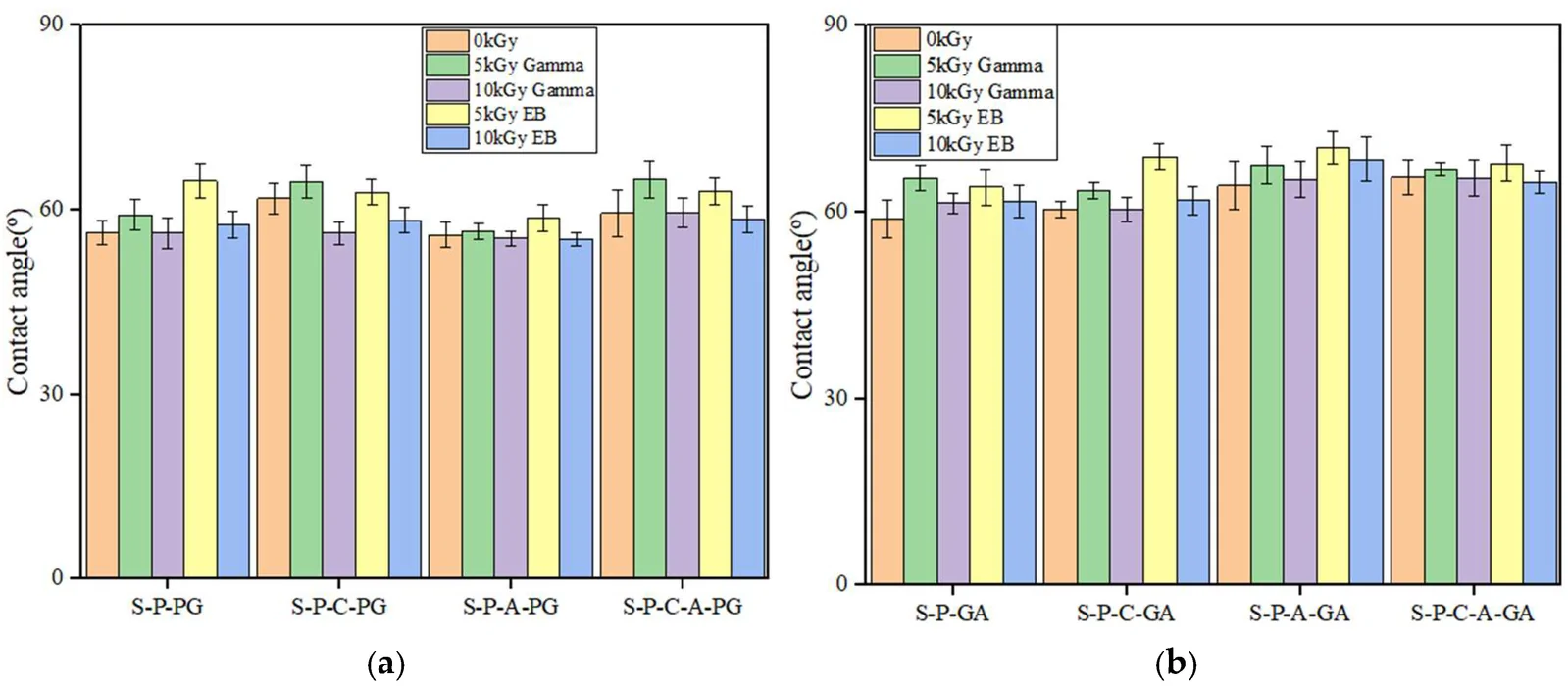
Water contact angle of (**a**) PG films and (**b**) GA films.

All films were hydrophilic, with contact angles of 55–70°. Across formulations and irradiation conditions, GA films had higher contact angles than the corresponding PG films (Figure 5; additive main effect: 0 kGy, F(1,16) = 11.7, *p* = 0.004; 5 and 10 kGy γ, and 5 and 10 kGy EB, *p* ≤ 0.0002). This effect was consistent across formulations (interaction, *p* ≥ 0.07). Individual comparisons were significant only for S-P-A (5 and 10 kGy γ; −9.1° and −6.1°, *p* = 0.023 and 0.034) and S-P-C (10 kGy γ; −9.2°, *p* = 0.047), indicating that the difference between additives was modest—about 4–9°. Contact angle depends not only on the film surface but also on roughness, water absorption during measurement, and residual glycerol or ethanol, none of which was characterised. Surface composition was not analysed, so we cannot determine whether GA and PG differ in their distribution or orientation at the air-facing surface. Further analysis, such as XPS, ATR-FTIR of both surfaces, or surface free-energy analysis, would be needed to explore this possibility. The results also show that wettability and bulk water uptake do not follow the same trend: despite their higher contact angles, S-P-A-GA and S-P-C-GA swelled more than the corresponding PG films (Section 3.2).

Irradiation significantly affected the overall contact angle for both additives (PG, F(4,40) = 13.3; GA, F(4,40) = 8.9; both *p* < 0.001), but no clear formulation effect was observed (interaction, *p* = 0.067 for PG and *p* = 0.38 for GA). Few individual comparisons with the non-irradiated control were significant: after 5 kGy EB, contact angles increased by 8.4° for S-P-PG and 8.5° for S-P-GA (*p* = 0.010 and 0.001, respectively). The remaining formulations showed non-significant increases of 1–7° at 5 kGy. The dose effect was consistent: at 5 kGy, contact angles were higher than at 10 kGy in all four PG formulations (*p* = 0.034 for S-P-A, *p* ≤ 0.008 for the others) and in S-P-GA and S-P-A-GA (*p* = 0.002 and 0.048, respectively). At 10 kGy, values returned to near those of the non-irradiated films (for example, S-P-C-PG was 64.59° at 5 kGy γ and 56.23° at 10 kGy γ). At 5–10 kGy, EB-irradiated films had higher contact angles than γ-irradiated films only for S-P (PG, *p* = 0.042; GA, *p* = 0.013). The increase at 5 kGy and the return to near non-irradiated values at 10 kGy remain unexplained, as neither surface chemistry nor roughness was measured.

### 3.5. Gas Chromatography Analysis

Gas evolution was measured in γ-irradiated films at 28 kGy (Table 6 and Table 7; n = 3). H_2_ yields ranged from 0.078 to 0.107 µmol J^−1^ for PG films and from 0.042 to 0.067 µmol J^−1^ for GA films. O_2_ yields were 0.084–0.098 and 0.065–0.072 µmol J^−1^, respectively. The only consistent difference was between additives: PG-containing films produced more H_2_ and O_2_ than their corresponding GA-containing films across all four formulations. However, because the additives were loaded at equal weight rather than equal moles, and no unloaded films were measured, this difference cannot be attributed to the additive alone. Alginate’s effect depended on the additive: H_2_ yields were higher in S-P-A-PG and S-P-C-A-PG than in S-P-PG and S-P-C-PG, but this pattern was not observed in the GA series. CNC showed no consistent trend in H_2_ or O_2_ beyond variation between replicates.

The observed differences may be related to radical chemistry, although the data do not identify the mechanism. One possibility is that the different phenolic structures (GA is more polar and carries a free carboxyl group, whereas PG has a hydrophobic propyl ester) affect how radicals formed in the polymer matrix are quenched or recombine, which may influence H_2_ yield. Another possibility is that alginate decarboxylation provides additional labile hydrogen in PG films. CNC may also restrict radical mobility. These explanations remain hypotheses: H_2_ and O_2_ yields alone cannot distinguish among hydrogen abstraction, phenoxyl-radical stabilisation, decarboxylation, radical trapping, or interfacial recombination. Testing these possibilities would require EPR, unloaded-film and radical-scavenger controls, product analysis (e.g., CO_2_, CO, HPLC-MS), and yield measurements across a range of doses.

### 3.6. Thermogravimetric Analysis

**Figure 6:**
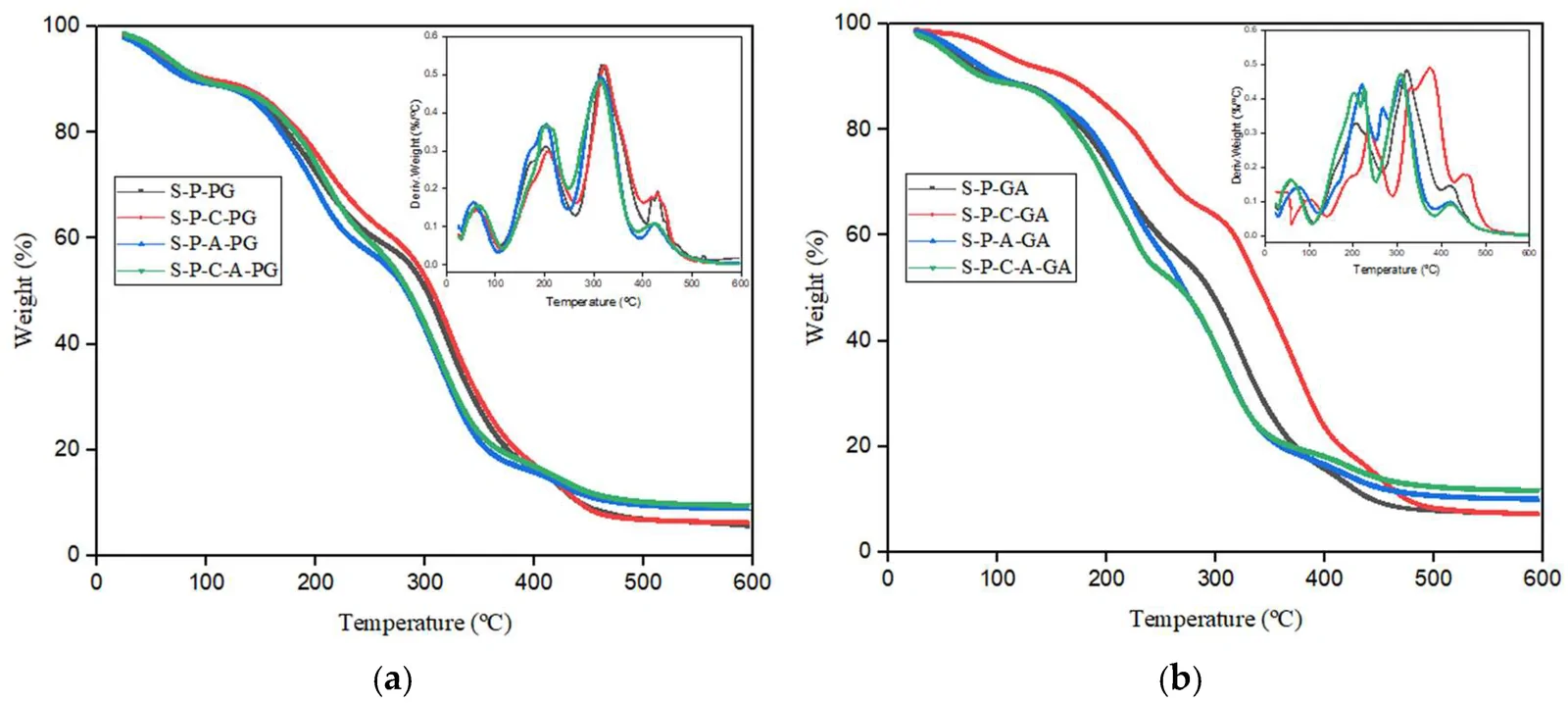
TGA curves of non-irradiated samples: (**a**) PG samples and (**b**) GA samples.

**Figure 7:**
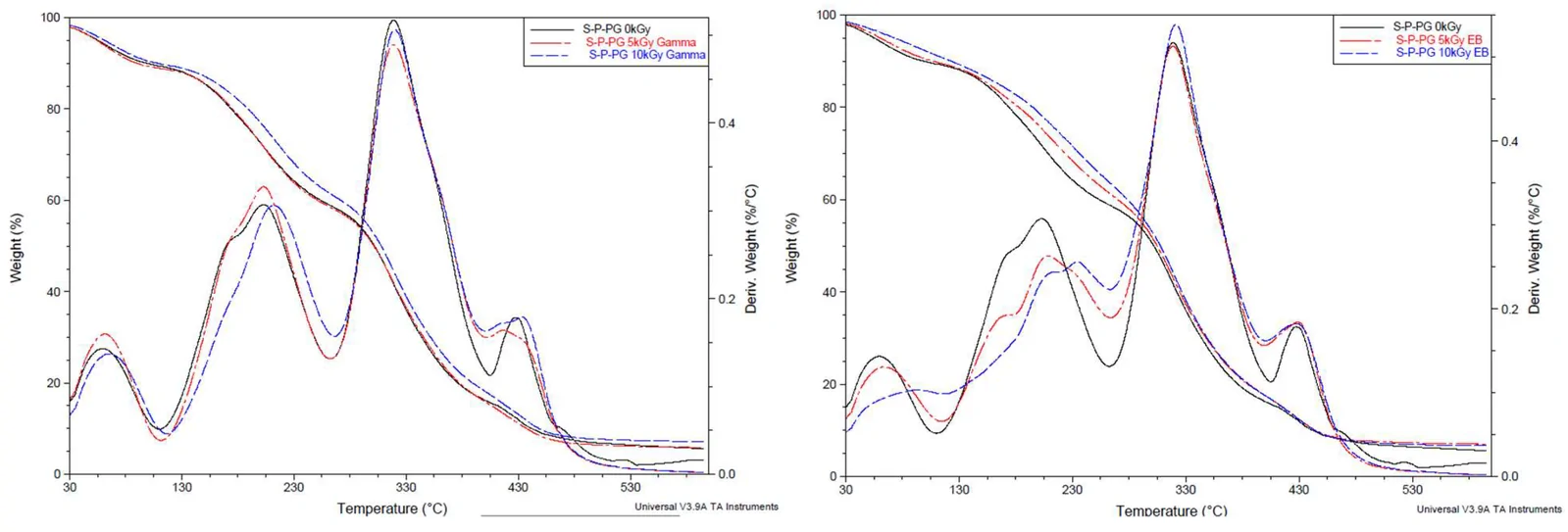
TGA curves of non-irradiated and irradiated samples for S-P-PG.

**Figure 8:**
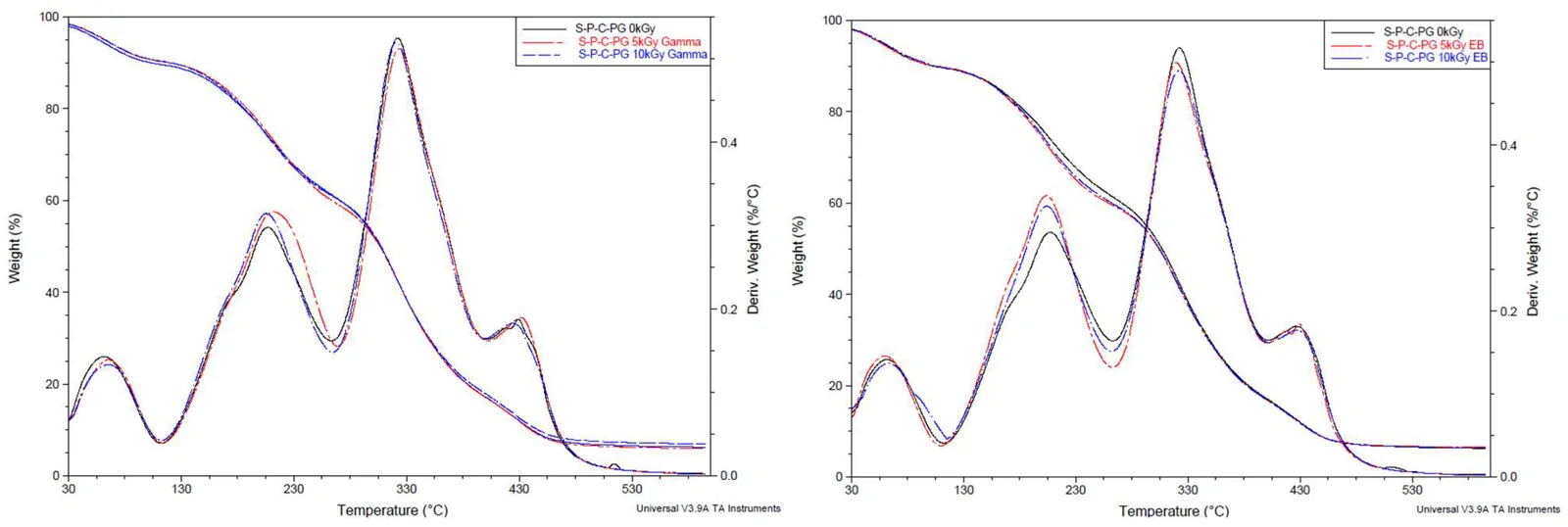
TGA curves of non-irradiated and irradiated samples for S-P-C-PG.

**Figure 9:**
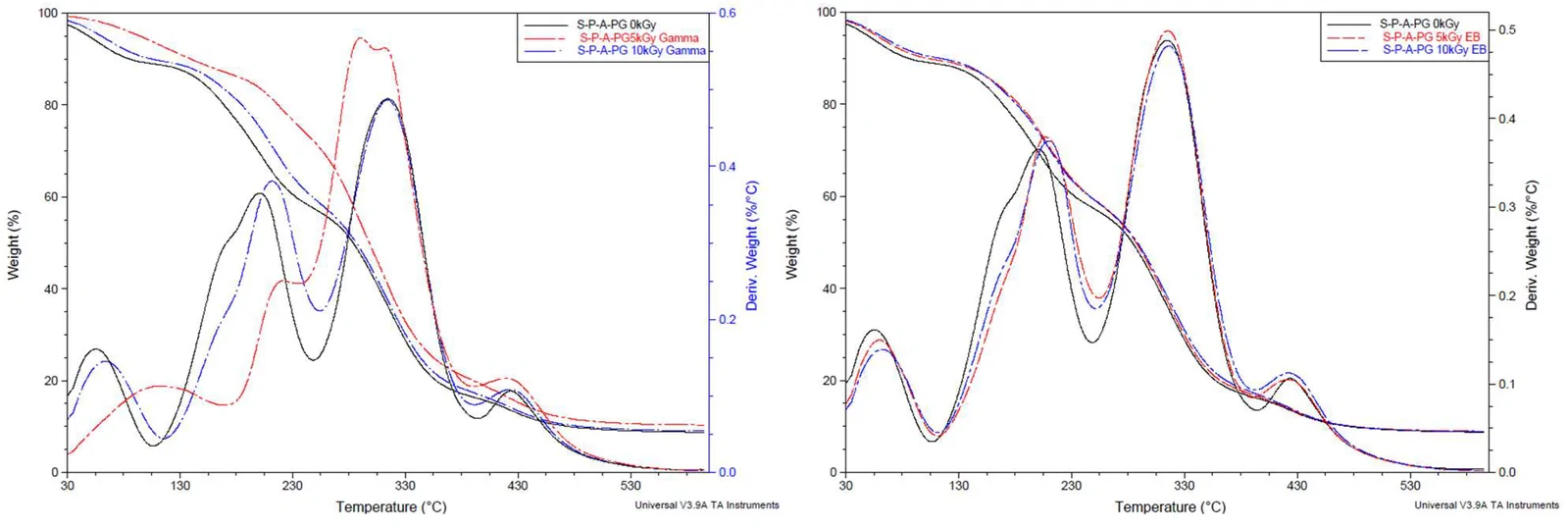
TGA curves of non-irradiated and irradiated samples for S-P-A-PG.

**Figure 10:**
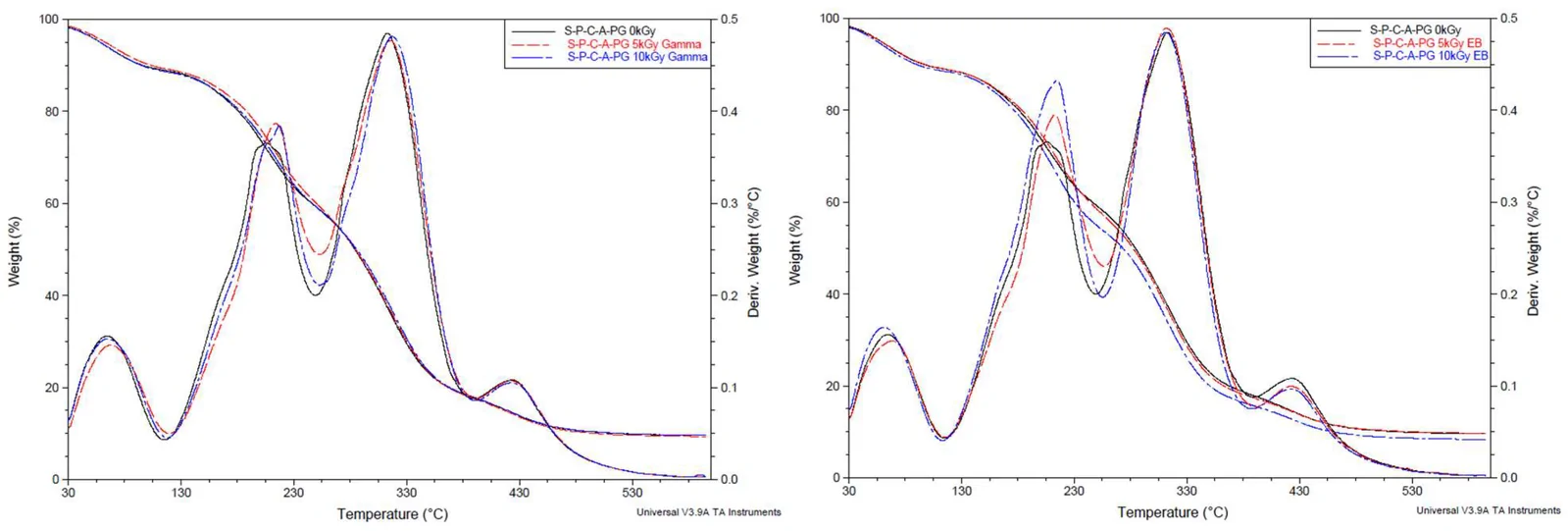
TGA curves of non-irradiated and irradiated samples for S-P-C-A-PG.

**Figure 11:**
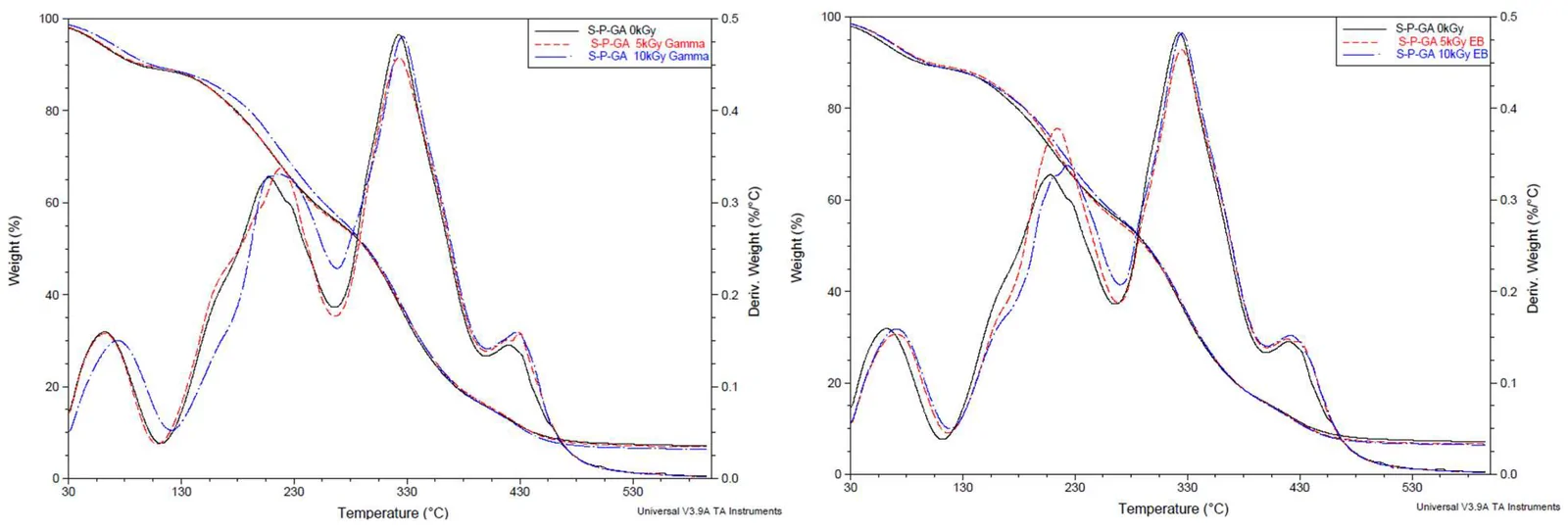
TGA curves of non-irradiated and irradiated samples for S-P-GA.

**Figure 12:**
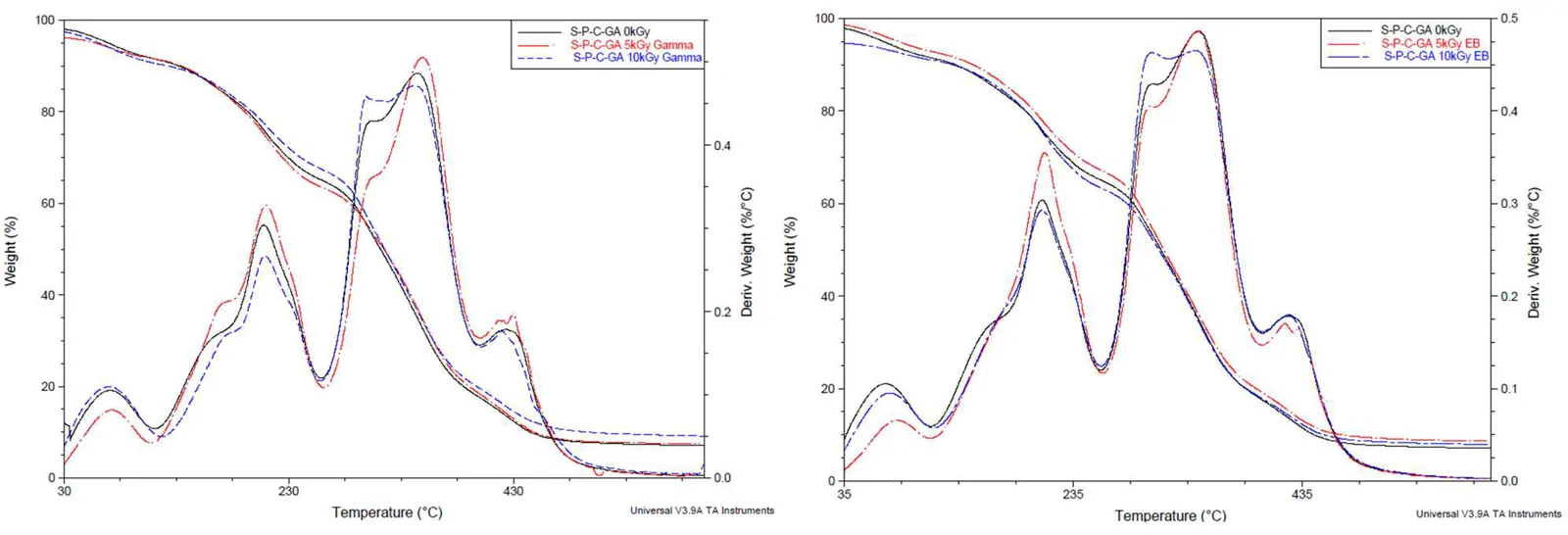
TGA curves of non-irradiated and irradiated samples for S-P-C-GA.

**Figure 13:**
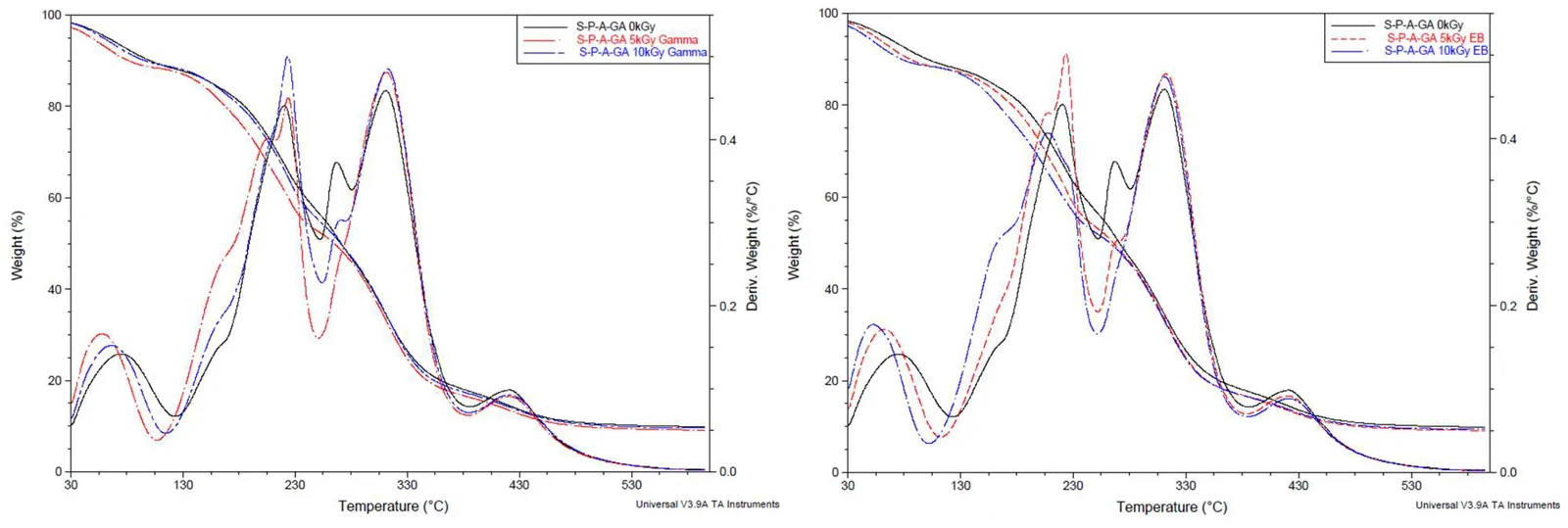
TGA curves of non-irradiated and irradiated samples for S-P-A-GA.

**Figure 14:**
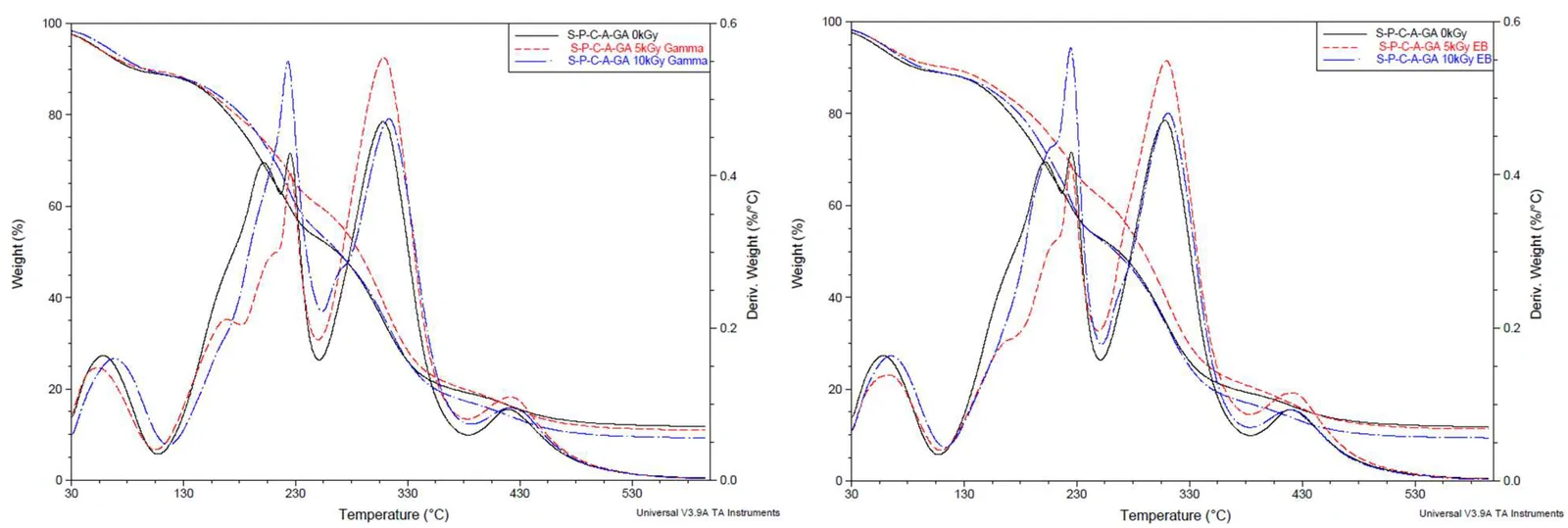
TGA curves of non-irradiated and irradiated samples for S-P-C-A-GA.

The TGA curves of the non-irradiated and irradiated films (Figure 6, Figure 7, Figure 8, Figure 9, Figure 10, Figure 11, Figure 12, Figure 13 and Figure 14) showed the typical multi-stage mass loss seen in starch–PVA-based films: sorbed water was lost below about 120 °C, followed by a main decomposition stage between about 200 and 350 °C, attributed to starch, PVA, and alginate [52,53]. At higher temperatures, the films lost mass more gradually, leaving a residue. Characteristic temperatures (T_onset, T_max, and T_final) are listed in Table 8 and Table 9. Because each value comes from a single run, no standard deviation is available. The temperature resolution is about 0.6 °C, so differences of just a few degrees may fall within run-to-run variation. For non-irradiated films, T_max ranged from 308.22 to 345.93 °C. CNC markedly increased T_max in GA films (S-P-GA 323.54 to S-P-C-GA 345.93 °C), but the increase was smaller in PG films (318.43 to 322.40 °C). Alginate lowered both T_max and T_final: T_max fell from 323.54 to 311.63 °C in GA films and from 318.43 to 313.90 °C in PG films, while T_final fell from 468.17 to 448.88 °C and from 479.51 to 461.93 °C, respectively. These results indicate that alginate did not improve thermal stability in this system. The T_max of S-P-C-A films (308.22 °C for GA, 313.90 °C for PG) was also lower than that of S-P-C films, suggesting that combining CNC and alginate did not increase thermal stability. Restricted chain mobility due to CNC may help explain its effect in GA films, although this possibility was not tested and the effect was not observed in the PG series.

T_onset was 2.5–5.7 °C higher in PG films than in the corresponding GA films across all four formulations (e.g., 197.80 vs. 192.13 °C for S-P). By contrast, T_max was higher for GA in S-P (323.54 vs. 318.43 °C) and S-P-C (345.93 vs. 322.40 °C), but higher for PG in S-P-A (313.90 vs. 311.63 °C) and S-P-C-A (313.90 vs. 308.22 °C). Thus, the data do not support a general conclusion that both additives increase thermal stability. GA’s greater hydrophilicity may explain its lower T_onset, since sorbed water and glycerol can affect the onset; however, this possibility was not tested.

The effects of 5–10 kGy irradiation were modest and did not show a consistent direction (Figure 7, Figure 8, Figure 9, Figure 10, Figure 11, Figure 12, Figure 13 and Figure 14). T_max changed by no more than 3.5 °C in seven of the eight formulations. The exception was S-P-C-A-GA, which increased by 5.1 °C (from 308.22 to 313.33 °C at 10 kGy γ). T_onset increased in S-P-GA (by 5–11 °C under all irradiation conditions) and in S-P-PG at 10 kGy EB (+9.6 °C). It decreased in S-P-A-GA (by 3–10 °C) and in S-P-A-PG after EB irradiation (by 6 °C), while the remaining films showed no systematic change. At 5 kGy γ, T_onset also decreased in S-P-PG (−7.4 °C) and S-P-C-GA (−3.8 °C), then returned to or exceeded the non-irradiated value at 10 kGy γ. In contrast, S-P-C-A-PG increased by 4–7 °C. These non-monotonic changes fall within the resolution of a single run. Since T_onset depends on sorbed water and glycerol content, we have not interpreted these changes. T_final for S-P-PG was 7–12 °C lower after every irradiation treatment.

### 3.7. Phenolic Content and Antioxidant Activity

**Figure 15:**
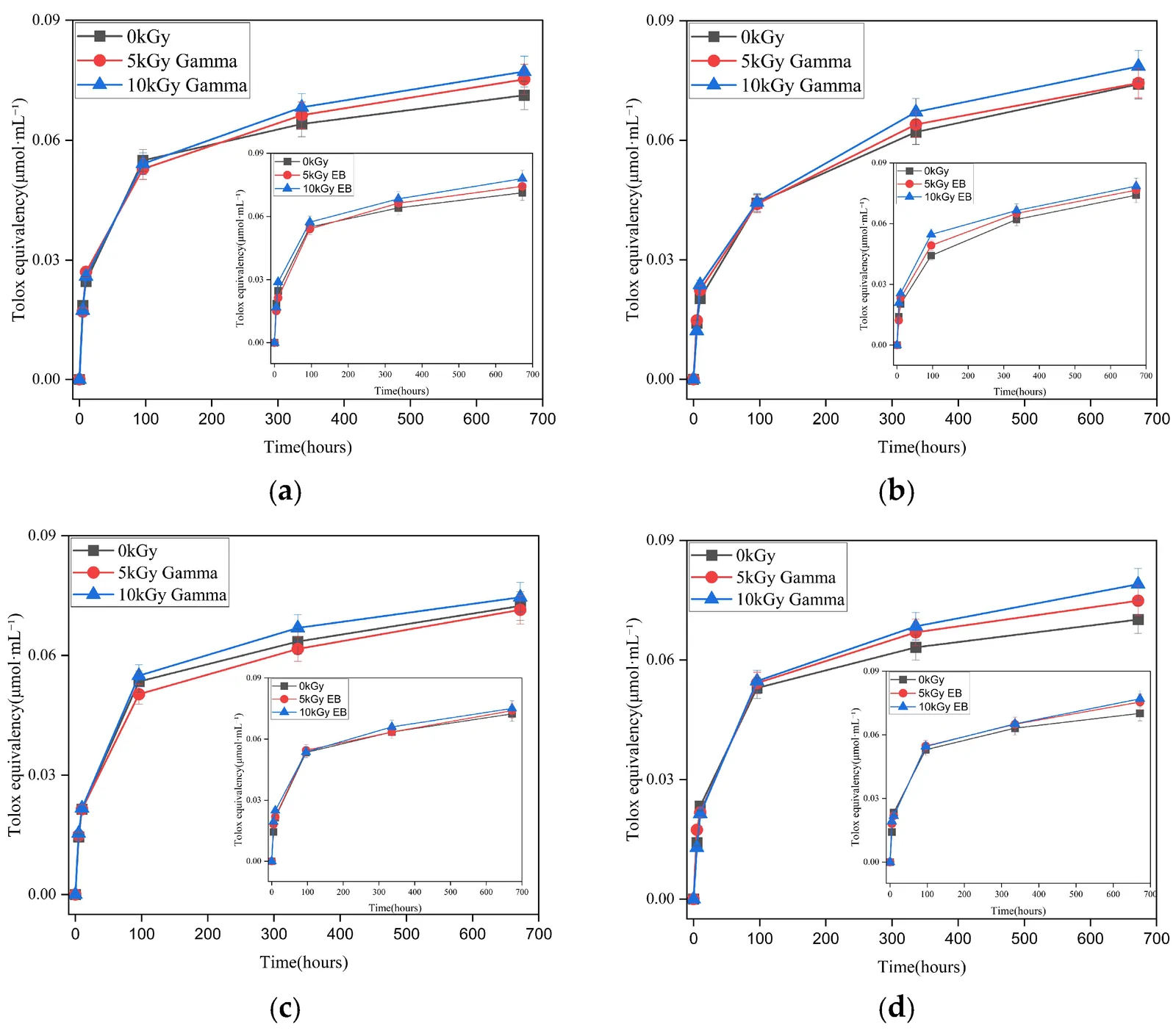
Trolox equivalent antioxidant capacity (TEAC) of ethanol extracts of PG-loaded films after 0–672 h of extraction. Main panels: γ-irradiated films; insets: EB-irradiated films (0, 5 and 10 kGy). (**a**) S-P-PG; (**b**) S-P-C-PG; (**c**) S-P-A-PG; (**d**) S-P-C-A-PG. Mean ± SD, n = 3.

**Figure 16:**
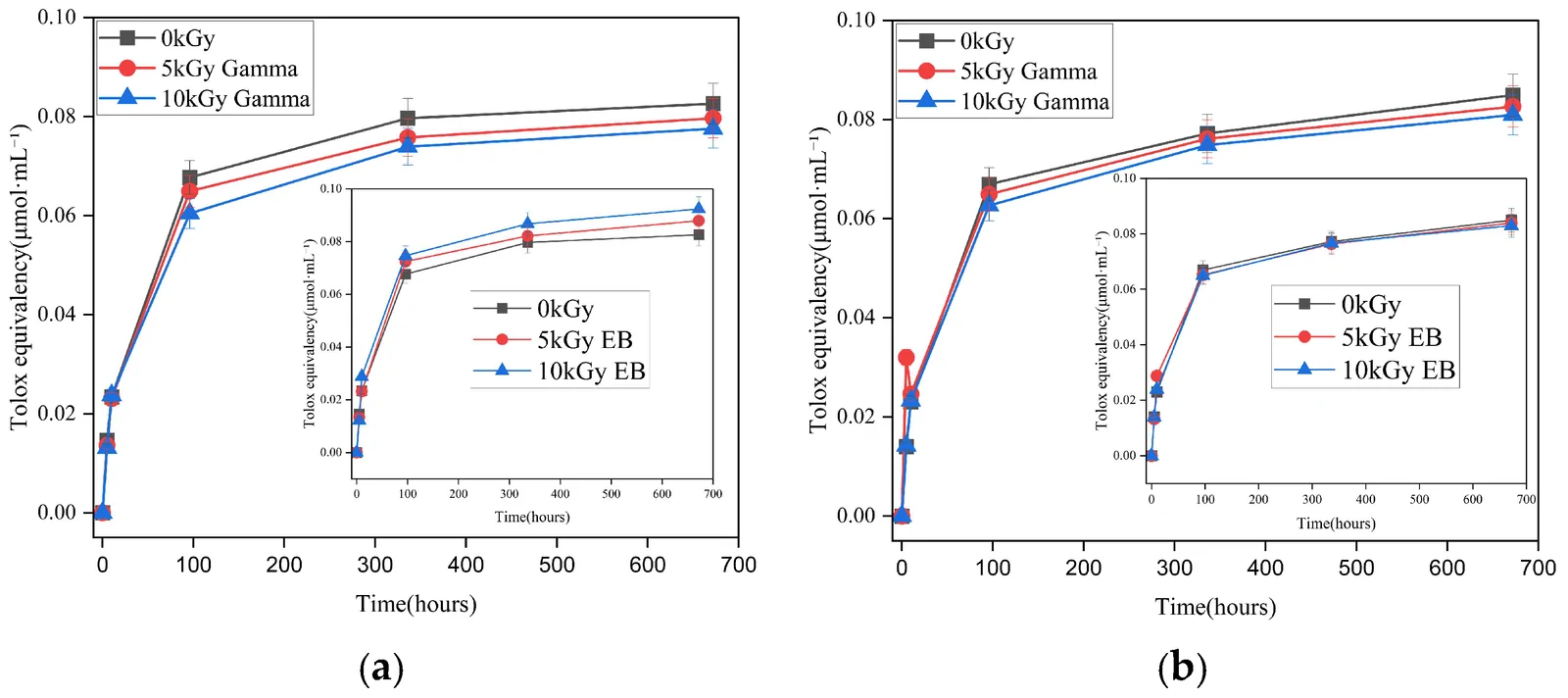

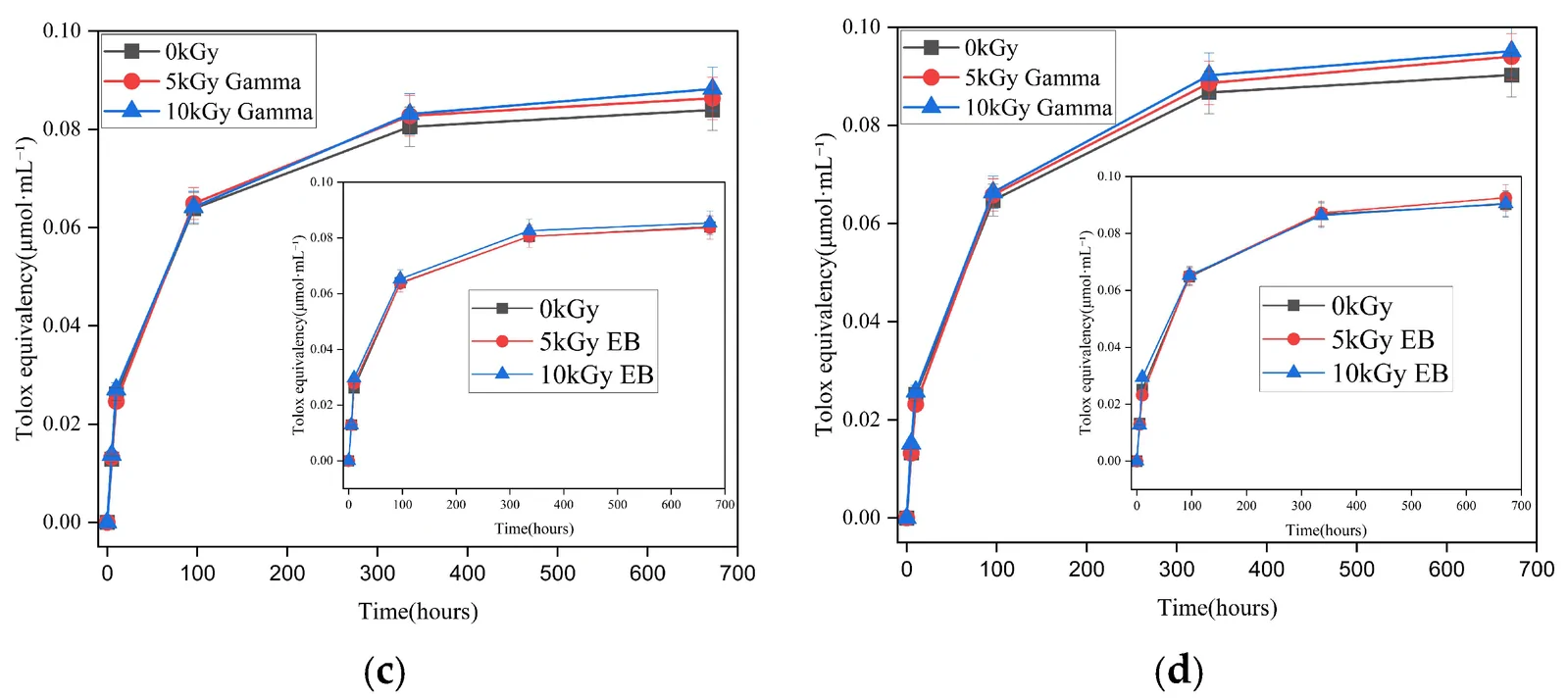
Trolox equivalent antioxidant capacity (TEAC) of ethanol extracts of GA-loaded films after 0–672 h of extraction. Main panels: γ-irradiated films; insets: EB-irradiated films (0, 5 and 10 kGy). (**a**) S-P-GA; (**b**) S-P-C-GA; (**c**) S-P-A-GA; (**d**) S-P-C-A-GA. Mean ± SD, n = 3.

TEAC values for the ethanol extracts changed little with irradiation (Figure 15 and Figure 16). After 672 h of extraction, the 10 kGy γ curves for PG films were about 4–11% higher than those of the non-irradiated controls, with the smallest difference in S-P-A-PG and the largest in S-P-C-A-PG. For GA films, the 10 kGy γ curves were about 5–6% lower than the controls in S-P-GA and S-P-C-GA, and about 5% higher in S-P-A-GA and S-P-C-A-GA. Thus, the direction of change varied by formulation and additive, while the differences remained small. Several explanations are possible, although none were tested. Irradiation altered swelling, solubility, and the insoluble fraction (Section 3.2 and Section 3.3), which may have affected the extraction efficiency of the additives. Oxidised or hydroxylated gallates, as well as dimeric or oligomeric products, could remain in the extract and still react with DPPH, helping preserve TEAC even if the intact additive was reduced. Soluble polymer fragments and reducing end groups released by irradiation may also react with DPPH. Covalent binding of the additives to the polymer would reduce, rather than preserve, the activity of the extract because the bound species would remain in the film; this possibility was not examined. Distinguishing among these explanations would require HPLC or LC-MS analysis of the extracts, an antioxidant mass balance (extracted, remaining, and degraded), analysis of exhaustively washed films, and matrix controls. The additive comparison is also influenced by equal-weight loading (GA has about 25% more moles per unit mass), so TEAC values for the two additives should be compared on a molar basis.

## 4. Conclusions

In starch–PVA films prepared from gamma-pre-irradiated starch, the effects of cellulose nanocrystals (CNCs, 1%), sodium alginate (5%), and the phenolic additive on the measured properties were interdependent. Propyl gallate (PG) produced greater tensile strength than gallic acid (GA) in the unfilled, alginate-containing, and CNC-containing films. All films remained hydrophilic, with contact angles of 55–70°. Irradiation at 5–10 kGy (γ or electron beam) largely preserved tensile properties, except for a 14–22% reduction in strength in S-P-C-A-GA. It reduced the insoluble fraction by up to 24% in six of the eight films and decreased swelling primarily in CNC-containing films, indicating a net loss of retained material. The antioxidant capacity of the ethanol extracts changed little after irradiation, and at 28 kGy γ, PG films had higher H_2_ and O_2_ yields than GA films. These observations may reflect a physically associated CNC/alginate network, chain scission or disruption of physical associations following irradiation, and differences in the radical chemistry and extractability of GA and PG. These explanations were not tested in this study. Hydrogen-bond bridging, CNC percolation, radical stabilisation, alginate decarboxylation, and the formation of transformed or polymer-bound antioxidant species remain hypotheses. The study also has several limitations: the films were prepared from pre-irradiated starch; each formulation was produced in a single casting batch; and CNC and alginate were each tested at only one concentration. The insoluble fraction is an operational measure obtained after hot-water treatment, and neither covalent crosslinking nor chain scission was measured directly. Gas evolution was measured at only one dose and with one modality. Antioxidant capacity was measured in ethanol extracts and does not represent migration into food. No SEM, FTIR, product identification, barrier (water vapour or oxygen), optical, migration, food-contact safety, biodegradation, or food-preservation data were obtained. Accordingly, packaging performance was not assessed, and no shelf-life effect is claimed. Future work could help address these gaps by including unloaded-film and native-starch controls, replicate casting batches, molecular-weight and spectroscopic analyses, SEM, HPLC-MS analysis of extracts and exhaustively washed films, barrier and migration tests in food simulants, biodegradation tests, and a food-preservation trial.

## Figures and Tables

**Table 1:** Formulation of the starch–PVA films (per batch): component masses (g) and percentage of total polymer solids.

Component	Starch-PVA (S-P)	Starch-PVA-Alginate (S-P-A)	Starch-PVA-CNC (S-P-C)	Starch-PVA-CNC-Alginate (S-P-C-A)
Starch (g; % of polymer)	8.655; 45	11; 55	8.9; 44.5	7.9; 39.5
PVA (g; %)	10.655; 55	8; 40	10.9; 54.5	10.9; 54.5
CNC (g; %)	0	0	0.2; 1	0.2; 1
Sodium alginate (g; %)	0	1; 5	0	1; 5
**Total polymer (g)**	20	20	20	20
Glycerol (% of polymer)	30	30	30	30
PG or GA (g; % of polymer)	0.69; 3.45	0.69; 3.45	0.69; 3.45	0.69; 3.45
Ethanol (mL)	5	5	5	5

**Table 2:** Mechanical test of propyl gallate films for irradiated and non-irradiated samples.

	S-P-PG		S-P-A-PG		S-P-C-PG		S-P-C-A-PG	
*Doses*	TS	EAB	TS	EAB	TS	EAB	TS	EAB
0 kGy	12.46 ± 1.18	187.88 ±18.21	16.88 ± 0.66	325.16 ± 20.69	15.17 ± 1.51	279.77 ± 21.31	13.40 ± 1.74	274.05 ± 12.07
5 kGy γ	12.00 ± 1.28	194.57 ± 10.46	16.85 ± 1.87	338.63 ± 10.37	15.18 ± 0.67	293.45 ± 10.04	13.78 ± 2.39	283.81 ± 18.29
10 kGy **γ**	12.92 ± 1.93	203.01 ± 27.86	16.11 ± 1.97	327.09 ± 19.12	15.01 ± 1.52	298.85 ± 15.64	13.46 ± 2.18	291.13 ± 10.04
5 kGy EB	13.15 ± 1.55	207.08 ± 28.96	15.73 ± 1.56	328.00 ± 5.47	15.60 ± 1.73	331.25 ± 17.05	13.89 ± 1.91	275.22 ± 8.77
10 kGy EB	12.03 ± 1.41	187.48 ± 13.58	17.21 ± 2.17	374.24 ± 17.72	15.83 ± 1.06	330.57 ± 23.82	11.19 ± 2.10	290.96 ± 9.80

**Table 3:** Mechanical test of gallic acid films for irradiated and non-irradiated samples.

	S-P-GA		S-P-A-GA		S-P-C-GA		S-P-C-A-GA	
Doses	TS	EAB	TS	EAB	TS	EAB	TS	EAB
0 kGy	9.30 ± 0.79	178.77 ± 5.88	11.29 ± 0.90	359.84 ± 36.22	9.95 ± 0.99	190.90 ± 7.33	14.36 ± 0.61	506.01 ± 19.93
5 kGy γ	9.44 ± 0.88	183.86 ± 12.37	11.07 ± 0.67	365.01 ± 37.35	9.22 ± 0.13	200.71 ± 5.76	13.95 ± 0.58	495.10 ± 20.94
10 kGy **γ**	9.72 ± 0.21	189.56 ± 11.23	10.20 ± 0.76	396.56 ± 20.52	10.08 ± 0.93	212.18 ± 17.06	12.40 ± 0.18	486.47 ± 31.55
5 kGy EB	9.84 ± 0.50	173.43 ± 13.70	11.36 ± 0.71	354.03 ± 17.87	9.90 ± 0.94	221.11 ± 15.47	11.77 ± 0.49	453.57 ± 20.46
10 kGy EB	9.60 ± 0.51	189.11 ± 22.70	11.03 ± 0.67	400.97 ± 32.08	10.26 ± 0.35	211.25 ± 19.32	11.26 ± 0.60	429.73 ± 36.84

**Table 4:** Two-way ANOVA (formulation × irradiation condition) of TS and EAB, by additive (df error = 140; n = 8 specimens per cell).

Response	Additive	Effect	F (df)	*p*	Partial η^2^
**TS**	PG	Formulation	45.0 (3,140)	<0.001	0.49
		Condition	0.40 (4,140)	0.81	0.01
		Interaction	1.54 (12,140)	0.12	0.12
**TS**	GA	Formulation	183.6 (3,140)	<0.001	0.80
		Condition	5.55 (4,140)	<0.001	0.14
		Interaction	11.6 (12,140)	<0.001	0.50
**EAB**	PG	Formulation	516.3 (3,140)	<0.001	0.92
		Condition	12.2 (4,140)	<0.001	0.26
		Interaction	6.28 (12,140)	<0.001	0.35
**EAB**	GA	Formulation	1518.6 (3,140)	<0.001	0.97
		Condition	3.50 (4,140)	0.009	0.09
		Interaction	7.54 (12,140)	<0.001	0.39

**Table 5:** Planned contrasts with Holm-adjusted *p* < 0.05 (irradiated minus 0 kGy, or EB minus γ at equal dose); other contrasts were not significant.

Response	Film	Contrast	Difference (95% CI)	Adj. *p*
TS (GA)	S-P-A-GA	10 kGy γ	−1.09 (−1.75, −0.43) MPa	0.018
	S-P-C-A-GA	10 kGy γ	−1.96 (−2.62, −1.30) MPa	<0.001
		5 kGy EB	−2.59 (−3.25, −1.93) MPa	<0.001
		10 kGy EB	−3.10 (−3.76, −2.44) MPa	<0.001
		EB vs. γ, 5 kGy	−2.18 (−2.84, −1.52) MPa	<0.001
		EB vs. γ, 10 kGy	−1.14 (−1.80, −0.48) MPa	0.006
EAB (PG)	S-P-A-PG	10 kGy EB	+49.1 (32.3, 65.9) %	<0.001
		EB vs. γ, 10 kGy	+47.2 (30.3, 64.0) %	<0.001
	S-P-C-PG	5 kGy EB	+51.5 (34.7, 68.3) %	<0.001
		10 kGy EB	+50.8 (34.0, 67.6) %	<0.001
		EB vs. γ, 5 kGy	+37.8 (21.0, 54.6) %	<0.001
		EB vs. γ, 10 kGy	+31.7 (14.9, 48.6) %	0.002
EAB (GA)	S-P-A-GA	10 kGy γ	+36.7 (14.4, 59.0) %	0.018
		10 kGy EB	+41.1 (18.8, 63.4) %	0.005
	S-P-C-A-GA	5 kGy EB	−52.4 (−74.7, −30.2) %	<0.001
		10 kGy EB	−76.3 (−98.6, −54.0) %	<0.001
		EB vs. γ, 5 kGy	−41.5 (−63.8, −19.2) %	0.002
		EB vs. γ, 10 kGy	−56.7 (−79.0, −34.5) %	<0.001

**Table 6:** Gas evolution of propyl gallate films.

	S-P-PG	S-P-C-PG	S-P-A-PG	S-P-C-A-PG
H_2_ (µmol/J)	0.078 ± 0.004	0.084 ± 0.005	0.107 ± 0.006	0.105 ± 0.004
O_2_ (µmol/J)	0.089 ± 0.005	0.084 ± 0.005	0.098 ± 0.005	0.090 ± 0.002

**Table 7:** Gas evolution of gallic acid films.

	S-P-GA	S-P-C-GA	S-P-A-GA	S-P-C-A-GA
H_2_ (µmol/J)	0.067 ± 0.003	0.063 ± 0.002	0.042 ± 0.004	0.066 ± 0.003
O_2_ (µmol/J)	0.068 ± 0.004	0.072 ± 0.001	0.065 ± 0.005	0.066 ± 0.004

**Table 8:** TGA data of GA films for both irradiated and non-irradiated samples.

	S-P-GA			S-P-C-GA			S-P-A-GA			S-P-C-A-GA		
	T_on_ Set	T_Final_	T_max_	T_on_ Set	T_Final_	T_max_	T_on_ Set	T_Final_	T_max_	T_on_ Set	T_Final_	T_max_
0 kGy	192.13	468.17	323.54	192.59	471.46	345.93	194.97	448.88	311.63	193.60	450.58	308.22
5 kGy γ	197.24	471.57	323.54	188.76	465.33	348.19	187.57	440.38	311.63	191.56	450.02	308.22
10 kGy **γ**	202.91	472.14	326.37	196.90	473.71	342.39	191.56	448.78	312.19	192.70	447.75	313.33
5 kGy EB	198.37	469.30	325.40	192.70	468.17	344.52	187.57	452.86	312.76	192.13	453.99	309.93
10 kGy EB	201.77	469.31	325.24	195.57	476.05	343.71	185.23	452.29	312.19	192.70	448.88	312.19

**Table 9:** TGA data of PG films for both irradiated and non-irradiated samples.

	S-P-PG			S-P-C-PG			S-P-A-PG			S-P-C-A-PG		
	T_on_ Set	T_Final_	T_max_	T_on_ Set	T_Final_	T_max_	T_on_ Set	T_Final_	T_max_	T_on_ Set	T_Final_	T_max_
0 kGy	197.80	479.51	318.43	196.67	468.73	322.40	197.80	461.93	313.90	196.10	458.53	313.90
5 kGy γ	190.43	469.30	318.43	197.40	473.24	323.54	197.80	466.67	314.46	202.91	469.30	316.16
10 kGy **γ**	198.37	470.44	320.13	197.80	470.44	320.70	199.50	458.53	314.46	200.64	472.14	317.30
5 kGy EB	197.80	467.60	319.57	196.10	472.70	319.00	192.13	456.82	315.03	196.10	464.74	312.19
10 kGy EB	207.44	472.20	321.27	199.50	472.12	322.40	191.56	465.90	316.73	200.07	463.63	311.63

## Data Availability

Data are contained within the article. Any additional raw data can be obtained upon request.
